# Solvents and Supporting Electrolytes in the Electrocatalytic Reduction of CO_2_

**DOI:** 10.1016/j.isci.2019.07.014

**Published:** 2019-07-16

**Authors:** Maximilian König, Jan Vaes, Elias Klemm, Deepak Pant

**Affiliations:** 1 Institute of Chemical Technology, University of Stuttgart, Pfaffenwaldring 55, 70569 Stuttgart, Germany; 2 Separation & Conversion Technology, Flemish Institute for Technological Research (VITO), Boeretang 200, 2400 Mol, Belgium

**Keywords:** Catalysis, Organic Reaction, Electrochemical Engineering

## Abstract

Different electrolytes applied in the aqueous electrocatalytic CO_2_ reduction reaction (CO_2_RR) considerably influence the catalyst performance. Their concentration, species, buffer capacity, and pH value influence the local reaction conditions and impact the product distribution of the electrocatalyst. Relevant properties of prospective solvents include their basicity, CO_2_ solubility, conductivity, and toxicity, which affect the CO_2_RR and the applicability of the solvents. The complexity of an electrochemical system impedes the direct correlation between a single parameter and cell performance indicators such as the Faradaic efficiency; thus the effects of different electrolytes are often not fully comprehended. For an industrial application, a deeper understanding of the effects described in this review can help with the prediction of performance, as well as the development of scalable electrolyzers. In this review, the application of supporting electrolytes and different solvents in the CO_2_RR reported in the literature are summarized and discussed.

## Graphical Abstract

**Figure:**
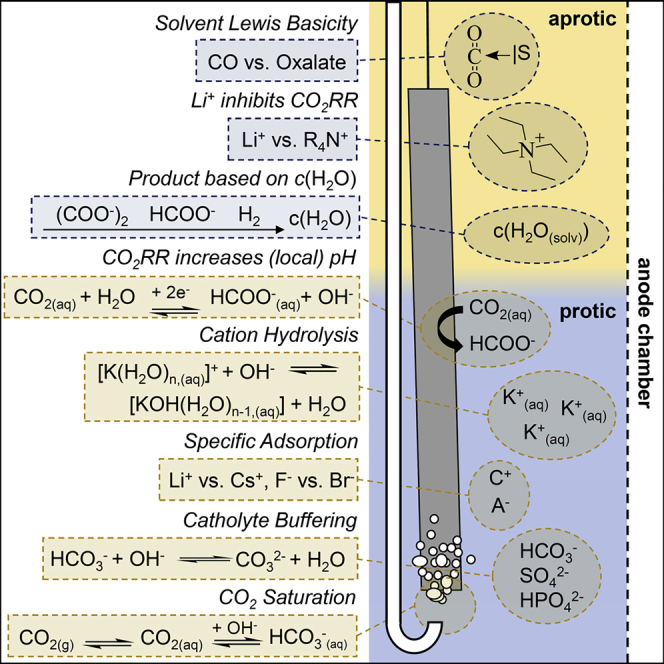

## Introduction

Carbon dioxide continues to accumulate in the atmosphere; the concentration is up by over 40% since the preindustrial era, from 280 ppm (parts per million) to 407 ppm today (August 2018, Mauna Loa Observatory, Hawaii; Recent Monthly CO_2_ Average Mauna Loa and Earth System Research Laboratory, 2018). The consumption of fossil fuels is the predominant reason for this increase in carbon dioxide in the Earth's atmosphere, which in turn is considered to be the major cause of climate change. To mitigate this effect, the European Union has committed to achieve an economy-wide domestic target of 80%–95% greenhouse gas reductions by 2050 compared with the 1990 levels (European Commission, 2011). This is needed to keep the temperature increase well below 2°C, as agreed to in the 2015 Paris Climate Agreement. In an effort to reduce the global CO_2_ emissions, a de-fossilization of our economy is inevitable (European Commision, 2018). This includes drastically reducing the use of fossil resources both as fuels in the energy sector and feedstocks in the chemical sector (Styring et al., 2015). With that initiative, new and sustainable technologies to produce fuels and chemicals with a carbon-neutral emission balance are desired. Carbon capture and utilization (CCU) technologies can contribute to the ultimate goal of closing the carbon cycle when using biomass-based CO_2_ (carbon-neutral) or fossil-based CO_2_ (CO_2_ recycling) to yield sustainable fuels (e.g., formic acid/formate for fuel cell applications, Bartrom and Haan, 2012), to produce commodity chemicals (e.g., ethylene), intermediates (e.g., oxalate), and final products (e.g., polymers). This includes the development of homogeneous (Finn et al., 2012, Windle and Perutz, 2012), heterogeneous, enzymatic (Shi et al., 2015), photo- (Taniguchi, 2012) and electrocatalytic (ElMekawy et al., 2016), as well as non-catalyzed thermal (Hu et al., 2013) processes. Some technologies are still in the early stages of development and are only present as a proof of concept or at the laboratory scale, whereas others are closer to market (e.g., CO_2_ to methanol, Styring et al., 2015; CO_2_ to methane, Audi E-Gas Project and Audi Technology Portal, 2013) or are already commercially applied (e.g., urea from CO_2_ and ammonia, Quadrelli et al., 2011).

With this in mind, the electrocatalytic reduction of CO_2_ is particularly promising, and research on this subject has increased rapidly in recent years. Consequently, review articles covering the electrocatalytic conversion of CO_2_ in general (Hori, 2008) and applied electrocatalysts (Gattrell et al., 2006, Hori, 2008, Bagger et al., 2017) and specific target products (Chaplin and Wragg, 2003, Lu et al., 2014) in particular are already available. However, most research in the electrocatalytic reduction of CO_2_ focuses on the development of the electrocatalyst to increase its selectivity, activity, and stability to reduce the cost of the final application. Although the catalyst is a key parameter in the development of an energy- and cost-efficient process to convert CO_2_ to products, many other factors need to be considered. These include, for example, the respective CO_2_ source (concentration, composition), the cell/electrolyzer design (batch versus flow conditions, Kopljar et al., 2016, Weekes et al., 2018; cell stacking), the chosen anode reaction (e.g., oxygen evolution reaction, Vermaas and Smith, 2016; alcohol oxidation, Lavacchi et al., 2014, Li et al., 2017, Wang et al., 2018; and chloride oxidation, Lister and Dufek, 2013), the supporting electrolyte of both reduction and oxidation reactions, the electrode engineering (e.g., gas diffusion electrodes [GDEs], Lai et al., 2018), applied membranes for conductivity and product separation (Vermaas and Smith, 2016), and the downstream processing (Greenblatt et al., 2018).

The aim of this review is to first give an overview of the solvents and electrolytes applied in the electrocatalytic reduction of CO_2_ and to then illustrate their impact on the electrochemical process. As both solvent and supporting electrolytes affect the CO_2_ reduction reaction (CO_2_RR) in multiple ways, their optimization is a crucial part of the development of a technically feasible and economically competitive CO_2_ electrolyzer. To achieve this, understanding of the effects influencing the efficiency of the electrochemical process is imperative. This review gathers the relevant parameters of reported solvent/electrolyte systems and in detail assesses their impact on the efficiency and the product distribution of the process, the latter determining the operational cost of further downstream processing. Although several publications have reported the systematic screening and optimization of solvents and electrolytes for different electrochemical systems, it has often proven difficult to individually tune the relevant parameters and ascribe them directly to the cell performance (e.g., faradaic efficiency [*FE*], energy efficiency [*EE*], limiting current density [*i*_limit_]). Besides the solubility of CO_2_ in the applied solvent, pH and buffering capacity, conductivity, toxicity, price, potential-current process window (stability), nature, and concentration of cation and anion species are other possibly relevant aspects to consider when choosing an appropriate solvent and electrolyte.

## Aqueous Electrocatalytic CO_2_ Reduction

### Solubility of CO_2_

Many research groups have discussed the supporting electrolyte as a significant factor in the effort to optimize the efficiency of the electrocatalytic reduction. Commonly applied aqueous electrolytes in the CO_2_RR include alkali (Singh et al., 2016) and ammonium (Kyriacou and Anagnostopoulos, 1993) salts of borates (Resasco et al., 2018), (bi-)carbonates (Varela et al., 2016a, Varela et al., 2016b), halides (Wu et al., 2012), hydroxides (Dinh et al., 2018), (dihydrogen-, hydrogen-)phosphates (Kortlever et al., 2013), and (hydrogen-)sulfates (Hong et al., 2017). The applied electrolytes are readily water soluble and should be stable in the applied potential regimes only, supporting the ionic transport and electrode reactions.

A key challenge to improve the efficiency of the electrocatalytic reduction of CO_2_ is the relatively low solubility of carbon dioxide in water at standard conditions. Applying a flat electrode, a CO_2_-saturated (*c* = 35 mmol·L^−1^) aqueous solution yields a low *i*_limit_, leveling off at around 20 mA·cm^−2^ (Qiao and Zhan, 2016). At current densities above *i*_limit_, the reaction is mass transport limited and the diffusion of CO_2_ to the active sites of the electrocatalyst is not sufficient. As a consequence, water is reduced to hydrogen (hydrogen evolution reaction [HER]) in an aqueous medium instead. The commercial application of electrolyzers for the electrocatalytic reduction of CO_2_ demands, among other parameters, high current densities to minimize the geometrical electrode surface area and electrolyzer size to optimize the space-time yield of the reactor and minimize capital expense. To overcome the challenge of CO_2_-limiting current densities, caused by its limited solubility in aqueous solution, several solutions have been proposed. The application of GDEs in the electrocatalytic reduction of CO_2_ was introduced by Mahmood et al. (1987). They permit current densities of almost two orders of magnitude higher at the same overpotential compared with planar electrodes (Weng et al., 2018) by introducing gaseous CO_2_ into the electrolyte at the active site through a gas diffusion layer inside the GDE. Alternatively, the mass transport of CO_2_ can be improved by increasing its solubility by adjusting the pressure (in MeOH, 333 mA·cm^2^, *FE*(CO) > 85%, 40 atm at Cu wire, Saeki et al., 1995a, Saeki et al., 1995b, up to 68 atm, Li and Prentice, 1997) and the temperature (down to −30°C, in MeOH, Naitoh et al., 1993, Mizuno et al., 1995, Kaneco et al., 1998a, Kaneco et al., 1998b, Kaneco et al., 1998c, Oh et al., 2014). Owing to requirements regarding the current density for an industrial application, the continuous supply of CO_2_ applying GDEs is preferred over submerged electrodes. Table 1 shows the recent literature on aqueous CO_2_RR at GDEs and the electrolytes applied.

**Table 1:** Publications in Recent Literature (2009–2019) in the Aqueous CO_2_RR Applying Gas Diffusion Electrodes in Semi-batch or Continuous Electrochemical Reactors under Standard Conditions

Catalyst/GDE Support	Electrolyte	Potential *E*_WE_/V	Current Density *i*/mA·cm^−2^	Faradaic Efficiency *FE*/-	Cell Setup	Publication Year
**Product: Formate/Formic Acid**						

Sn/carbon paper	0.5 M KCl at pH 4	3 V (E_Cell_)	100	89%	Continuous	2010 (Whipple et al., 2010)
Sn/carbon paper	2 M KCl	−2 V versus SCE	50	60%–70%	Semi-batch	2011 (Agarwal et al., 2011)
Sn/carbon paper	0.5 M NaHCO_3_ at pH 8.3	−1.6 V versus NHE	27	70%	Continuous	2013 (Prakash et al., 2013)
Sn/carbon paper	0.1 M KHCO_3_ at pH 7	−1.7 V versus Ag/AgCl	10	90%	Continuous	2013 (Wu et al., 2013)
Sn/carbon black	0.5 M NaHCO_3_	−1.8 V versus Ag/AgCl	13	73%	Semi-batch	2014 (Wang, Dong and Yu, 2014b)
Sn/carbon black	0.5 M KHCO_3_	−1.8 V versus Ag/AgCl	17	79%	Semi-batch	2014 (Wang, Dong and Yu, 2014a)
Sn/carbon paper	0.45 M KHCO_3_ +0.5 M KCl	−1.63 V versus Ag/AgCl	40	70%	Continuous	2014 (Del Castillo, Alvarez-Guerra and Irabien, 2014)
Sn/carbon paper	0.5 M KHCO_3_	−1.7 V versus SCE	Not reported	80%	Semi-batch	2014 (Wu et al., 2014a, Wu et al., 2014b)
Sn/carbon paper	0.1 M KHCO_3_	1.2 V (E_Cell_)	3	64%	Continuous	2014 (Wu et al., 2014a, Wu et al., 2014b)
Sn/carbon black	0.1 M KHCO_3_ at pH 10	−1.57 V versus SHE	200	90%	Semi-batch	2014 (Kopljar et al., 2014)
Sn/carbon black	0.1 M KHCO_3_ at pH 10	−1.57 V versus SHE	200	90%	Semi-batch	2015 (Kopljar et al., 2015)
Sn/carbon black	0.5 M KHCO_3_	−2.0 V versus Ag/AgCl	22 (partial)	87%	Semi-batch	2015 (Wang et al., 2015)
PtRu alloy/carbon paper	0.5 M K_2_SO_4_ at pH 2	−0.82 V versus Ag/AgCl	143	96%	Continuous	2016 (Lu et al., 2016a, Lu et al., 2016b)
PtRu, Pb/carbon paper	0.5 M K_2_SO_4_ at pH 2-14	ca. −2 V versus Ag/AgCl	ca. 300	95%	Continuous	2016 (Lu et al., 2016a, Lu et al., 2016b)
SnO_2_/carbon black	1 M KHCO_3_ at pH 10	Not reported	400	75%	Semi-batch	2016 (Kopljar et al., 2016)
InSn alloy/carbon paper	0.1 M KHCO_3_	−1.2 V versus RHE	15	92%	Semi-batch	2017 (Lai et al., 2017)
Sn/Carbon paper	0.5 M Na_2_CO_3_ +0.5 M Na_2_SO_4_	−1.6 V versus Ag/AgCl	388	80%	Semi-batch	2017 (Sen et al., 2017)
CuS/Carbon paper	0.1 M KHCO_3_	−0.8 V versus RHE	20	80%	Semi-batch	2018 (Shinagawa et al., 2018)

**Product: Carbon Monoxide**						

Ag GDE (Covestro)	0.5–0.8 M K_2_SO_4_	ca. 1.8 V versus Ag/AgCl	30	90%	Continuous	2011 (Dufek et al., 2011)
Ag/carbon paper	1 M KCl	−1.7 V versus Ag/AgCl	90 (partial)	94%	Continuous	2013 (Jhong et al., 2013)
Ag/TiO_2_	1 M KOH	−1.8 V versus Ag/AgCl	101 (partial)	90%	Continuous	2014 (Ma et al., 2014)
Ag/carbon paper	0.5 M K_2_HPO_4_ +0.5 M KH_2_PO_4_ at pH 10	3 V (E_Cell_)	Up to 51 (partial)	Up to 80%	Continuous	2015 (Kim et al., 2015)
Ag/carbon black, carbon paper	1 M KOH	−2.2 V versus Ag/AgCl	280 (partial)	Not reported	Continuous	2016 (Kim et al., 2016)
Ag/carbon paper	0.5 M KHCO_3_	−1.45 V versus Ag/AgCl	50	60%	Continuous (bipolar membrane)	2016 (Li et al., 2016)
Ag/carbon nanotubes	1 M KOH	−0.75 V versus RHE	350	>95%	Continuous	2016 (Ma et al., 2016a, Ma et al., 2016b)
Ag/carbon paper	3 M KOH	−0.96 V versus RHE	343	Up to 100%	Continuous	2016 (Verma et al., 2016)
Au/carbon nanotubes	2 M KOH	−1.45 V versus Ag/AgCl	120	90%	Continuous	2018 (Verma et al., 2018)
Ag GDE (Covestro)	1.5 M KHCO_3_ at pH 7	5 V (E_Cell_)	300	80%	Continuous	2018 (Haas et al., 2018)
Au/Carbon paper	0.1 M KHCO_3_	−1.3 V versus Ag/AgCl	10	90%	Continuous	2019 (Ahangari et al., 2019)

**Product: Methanol**						

Cu_2_O/carbon paper	0.5 M KHCO_3_	−1.39 V versus Ag/AgCl	10	55%	Continuous	2016 (Albo and Irabien, 2016)

**Product: Ethylene**						

Cu_2_O, Cu/carbon paper	1 M KOH	−0.7 V versus RHE	150 (partial, C_2_H_4_), 48 (partial, EtOH)	46% (C_2_H_4_), 17% (EtOH)	Continuous	2016 (Ma et al., 2016a, Ma et al., 2016b)
Cu/carbon paper	0.1 M KBr	Not reported	170	57%	Continuous	2017 (Reller et al., 2017)
Cu/Graphite, carbon nanoparticles	7 M KOH	−0.55 V versus RHE	75–100	70%	Continuous	2018 (Dinh et al., 2018)
Cu/carbon paper	1 M KOH	−0.66 V versus RHE	653	62%	Continuous	2018 (Lv et al., 2018)
CuAg alloy/carbon paper	1 M KOH	−0.7 V versus RHE	300	60% (C_2_H_4_), 25% (EtOH)	Continuous	2018 (Hoang et al., 2018)

NHE, normal hydrogen electrode; RHE, reversible hydrogen electrode; SCE, saturated calomel electrode; SHE, standard hydrogen electrode.

**Figure 1:**
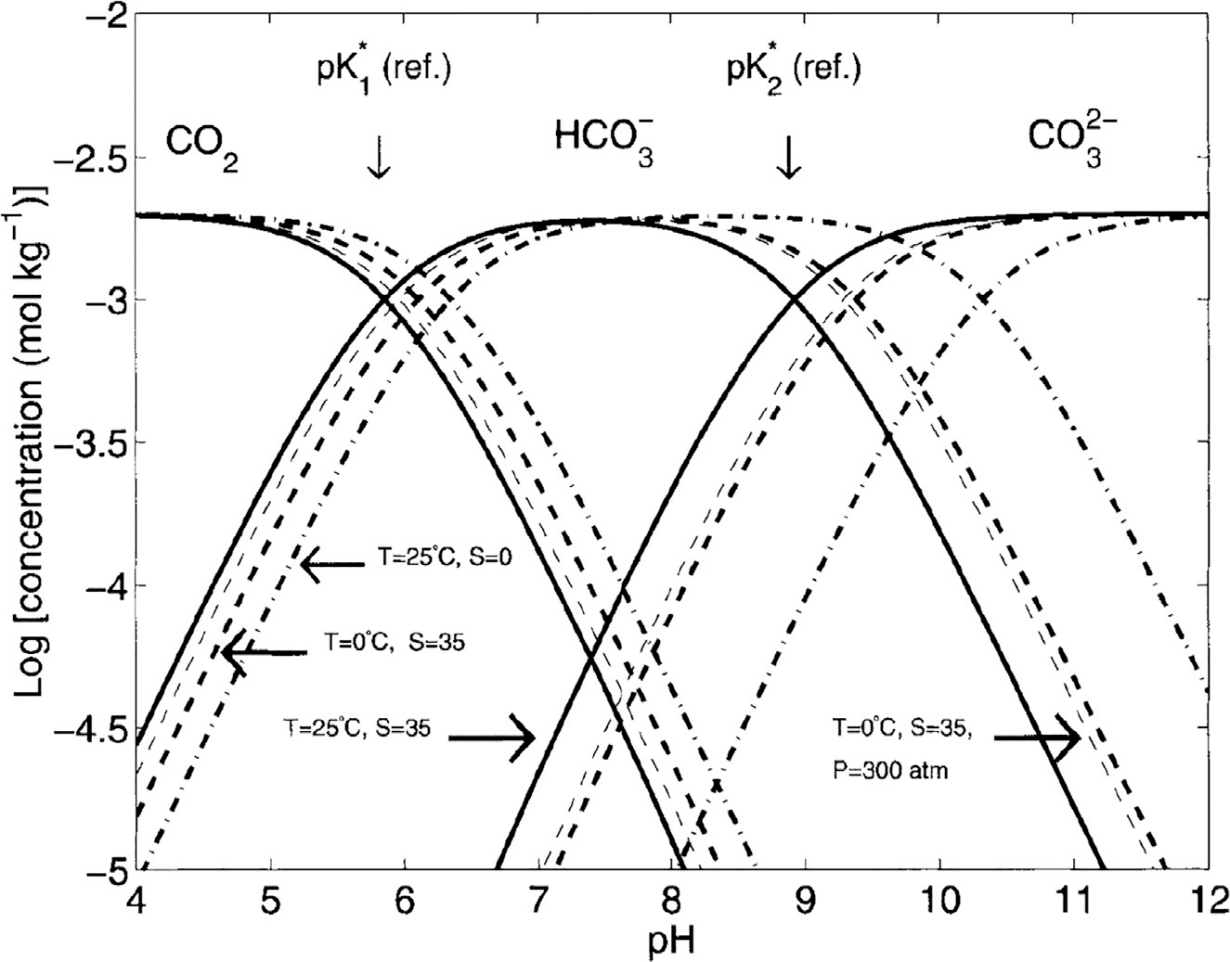
Solubility of CO_2_ in Water as a Function of the pH Value, At Indicated Temperature, Salinity, and Pressure Reference (solid line) at *T* = 25°C, *p* = 1 atm, and salinity *S* = 35 g·kg^−1^. Reproduced with permission from (Wolf-Gladrow and Zeebe, 2001).

Figure 1 depicts the dependency of the CO_2_ solubility on the pH of water and indicates speciation shifts with selected values of pressure, temperature, and salinity as described by Henry's law (Equation 1). It describes the relation between the solubility of an ideal gas in a solvent *c*(CO_2_) and the vapor pressure *p*_CO2_ of the gas over the solvent for diluted mixtures. *K*_CO2_ is the empirical Henry constant. *K*_CO2_ (in mol·atm·L^−1^) was fitted by Weiss (Weiss, 1974) to express the dependency on the absolute temperature *T* (in K) and the salinity *S* (in g·kg^−1^), see Equation 2.

(Equation 1) $$p_{CO2}=K_{CO2}\cdot c\left(CO_{2}\right)$$

(Equation 2) $$\ln\;K_{CO2}=-58.0931+90.5069\left(\frac{100}{T}\right)+22.2940\;\ln\left(\frac{T}{100}\right)+S\left[0.027766-0.023656\left(\frac{T}{100}\right)+0.0050578{\left(\frac{T}{100}\right)}^{2}\right]$$

The total amount of CO_2_ in solution is not limited to physically dissolved CO_2(aq)_ but is given by the total dissolved inorganic carbon (*DIC)* (Equation 3). The *DIC* includes the concentrations of carbonic acid, bicarbonate, and carbonate formed according to the carbonic acid equilibrium (see Equation 4). As H_2_CO_3_ constitutes less than 0.01% of the *DIC*, it is usually merged with CO_2(aq)_ to describe the total amount of CO_2_ in solution (Schulz et al., 2006).

(Equation 3) $$DIC=c\left(CO_{2}\left(aq\right)\right)+c\left(H_{2}CO_{3}\right)+c\left(HCO_{3}^{-}\right)+c\left(CO_{3}^{2-}\right)$$

(Equation 4) $$CO_{2\left(aq\right)}+H_{2}O\;\;⇄\;\;H_{2}CO_{3}\;\;⇄\;\;H^{+}+HCO_{3}^{2-}\;\;⇄\;\;2H^{+}+CO_{3}^{2-}$$

As indicated in Equation 4, the carbonic acid equilibrium is dependent on the pH value of the solution, shifting toward the formation of bicarbonate and carbonate with increasing pH values (see Figure 1). As dissolved CO_2_ is the main active species in the electrocatalytic reduction of CO_2_, the amount of electrochemically active CO_2_ in solution is decreased at higher pH values.

### Effect of (Local) pH Value

The pH value of the applied electrolyte depends on cation, anion species, and their respective concentrations. Both cations and anions impact the CO_2_RR in multiple ways; other observed effects besides the pH value are discussed in the following sections. This needs to be considered when comparing results of CO_2_RR experiments at different pH values. In addition to the effect the pH has on the CO_2_ solubility, the pH value impacts the thermodynamics of the CO_2_ reduction, according to the Nernst equation, see Equation 5. As H^+^ ions are consumed in the reaction, the activity of protons directly affects the equilibrium potential *E* of the reaction.

(Equation 5) $$E=E^{0}-\frac{R\cdot T}{z\cdot F}\cdot ln\frac{a_{Red}}{a_{Ox}}$$

**Figure 2:**
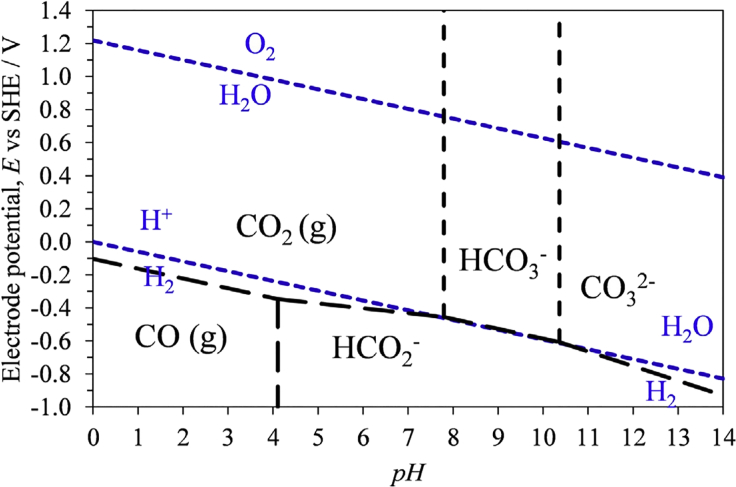
Meta-stable Potential-pH (Pourbaix) Diagram for a C-H_2_O System at 298 K and 1 Bar Reproduced with permission from (Bumroongsakulsawat and Kelsall, 2014).

The dependence of the standard potential to the pH value is typically displayed in Pourbaix diagrams (see Figure 2 for CO_2_/CO/HCOO^−^ system and the competing H_2_ evolution). In the reaction of each CO_2_RR product in aqueous media, water acts as a proton donor for the CO_2_RR product and/or intermediates. Acidic conditions not only facilitate the protonation but also favor the competing HER. Therefore, for both CO_2_RR and HER, based on the pH value in the solution, either OH^−^ is formed or H^+^ is consumed during the reaction (see Equations 6, 7, 8, 9, and 10, the standard electrode potentials are given versus standard hydrogen electrode at 25°C, pH 7, Hori, 2008, Kopljar et al., 2014).

(Equation 6) $$CO_{2}+H_{2}O+2\;e^{-}\;\;⇄\;\;HCOO^{-}+OH^{-}\;E^{0}=-0.43V$$

(Equation 7) $$CO_{2}+H_{2}O+2\;e^{-}\;\;⇄\;\;CO+2\;OH^{-}\;E^{0}=-0.52V$$

(Equation 8) $$CO_{2}+6\;H_{2}O+8\;e^{-}\;\;⇄\;\;CH_{4}+8\;OH^{-}\;E^{0}=-0.25V$$

(Equation 9) $$2\;CO_{2}+8\;H_{2}O+12\;e^{-}\;\;⇄\;\;C_{2}H_{4}+12\;OH^{-}\;E^{0}=-0.34V$$

(Equation 10) $$2\;CO_{2}+9\;H_{2}O+12\;e^{-}\;\;⇄\;\;C_{2}H_{5}OH+12\;OH^{-}\;E^{0}=-0.33V$$

The reduction of CO_2_ at low pH values is energetically favored as the required potential given by thermodynamics is reduced. Gao et al. (Gao et al., 2015) studied the electrocatalytic reduction of CO_2_ to CO on Pd nanoparticles in acidic media. They reported an increase in the FE for CO production with rising pH values from pH 1.5 to 4.2 at comparable potentials. The measured partial current density to CO passes through a maximum at pH 2.2, whereas the *FE* to CO is gradually increasing with the pH value, which is rationalized by a suppressed HER due to an increasing hydrogen binding energy at the catalyst surface, based on cyclic voltammetry measurements. Although not explicitly mentioned by the authors, the possibility of mass transport limitations through less available protons cannot be ruled out.

**Figure 3:**
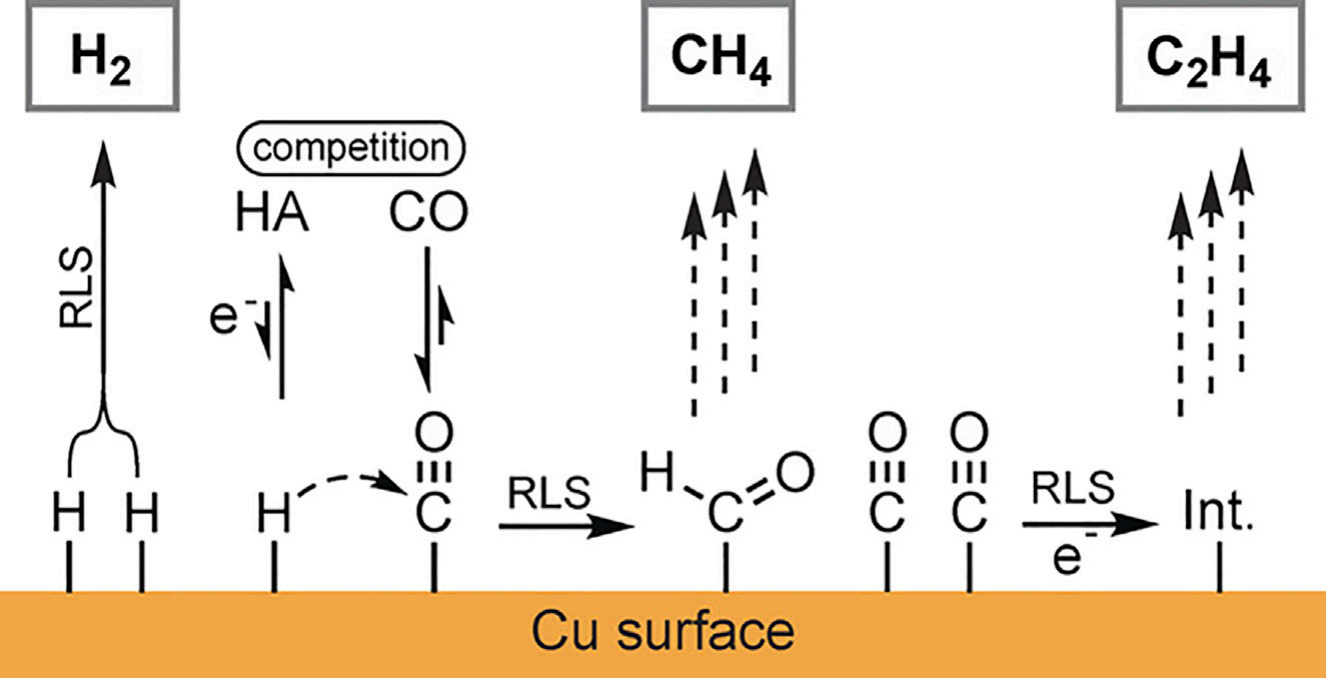
Proposed Mechanism for the Competitive Reduction Reactions of Water and CO_2_ at Cu to Form Hydrogen, Methane, and Ethylene RLS, rate-limiting step; Int., intermediate in the formation of ethylene. Reproduced with permission from (Schreier et al., 2018).

In the CO_2_RR on copper surfaces, the pH value has shown to impact the product distribution between methane (predominantly formed at low pH values) and C_2_ or longer reaction products such as ethylene C_2_H_4_, ethanol C_2_H_5_OH, and n-propanol C_3_H_7_OH (at larger pH values) (Hori et al., 1989). The rate-limiting step (RLS) for the formation of methane is dependent on the pH value, whereas the RLS in the formation of C_2+_ products is pH independent (Hori et al., 1997, Schouten et al., 2014). The formation of both methane and C_2+_ products proceeds through a metal-bound [M]-CO intermediate (see Figure 3). While the consequent protonation to form methane is dependent on the local proton concentration, the reaction mechanism for the formation of C_2+_ products is followed up by a rate-determining C-C coupling of two surface-bound [M]-CO species (Schreier et al., 2018). As the RLS of the CH_4_ formation is dependent on *c*(H^+^), the formation of C_2+_ products is favored over it at high pH values. In an attempt to maximize the *FE* toward C_2+_ products at Cu electrodes, Lum et al. (Lum et al., 2017) identified both the pH value and the local CO_2_ concentration as parameters that need to be optimized. Although the *FE*s to C_2+_ products increases with the pH value, a decrease in *FE* at strongly alkaline pH > 10 is reported. This is again related to the inactivation of CO_2_ to CO_3_^2−^ via the carbonic acid equilibrium and the competing HER.

Bumroongsakulsawat has investigated the effect of the pH value at Sn electrodes on the CO_2_RR product ratios of carbon monoxide and formate. Similar to publications discussed earlier, an increased HER was correlated with abundance of protons at low pH values (Bumroongsakulsawat and Kelsall, 2014). Concerning the production selectivity, expressed by means of the CO:HCOO^−^ ratio, a change from 1 to 0.15 was observed with a change from pH 2.9 (in 0.1 M H_3_PO_4_/1 M NaH_2_PO_4_) to pH 7.8 (in 0.5 M NaOH). This trend is in accordance with the standard potentials for the two products, which predict the favored formation of formate at pH > 4.1 (see Figure 2). Similar to the CO_2_RR at other electrocatalysts, Li et al. (Li and Oloman, 2006) reported a reduced efficiency to formate at pH > 9 on tin-coated copper mesh in a continuous reactor setup.

**Figure 4:**
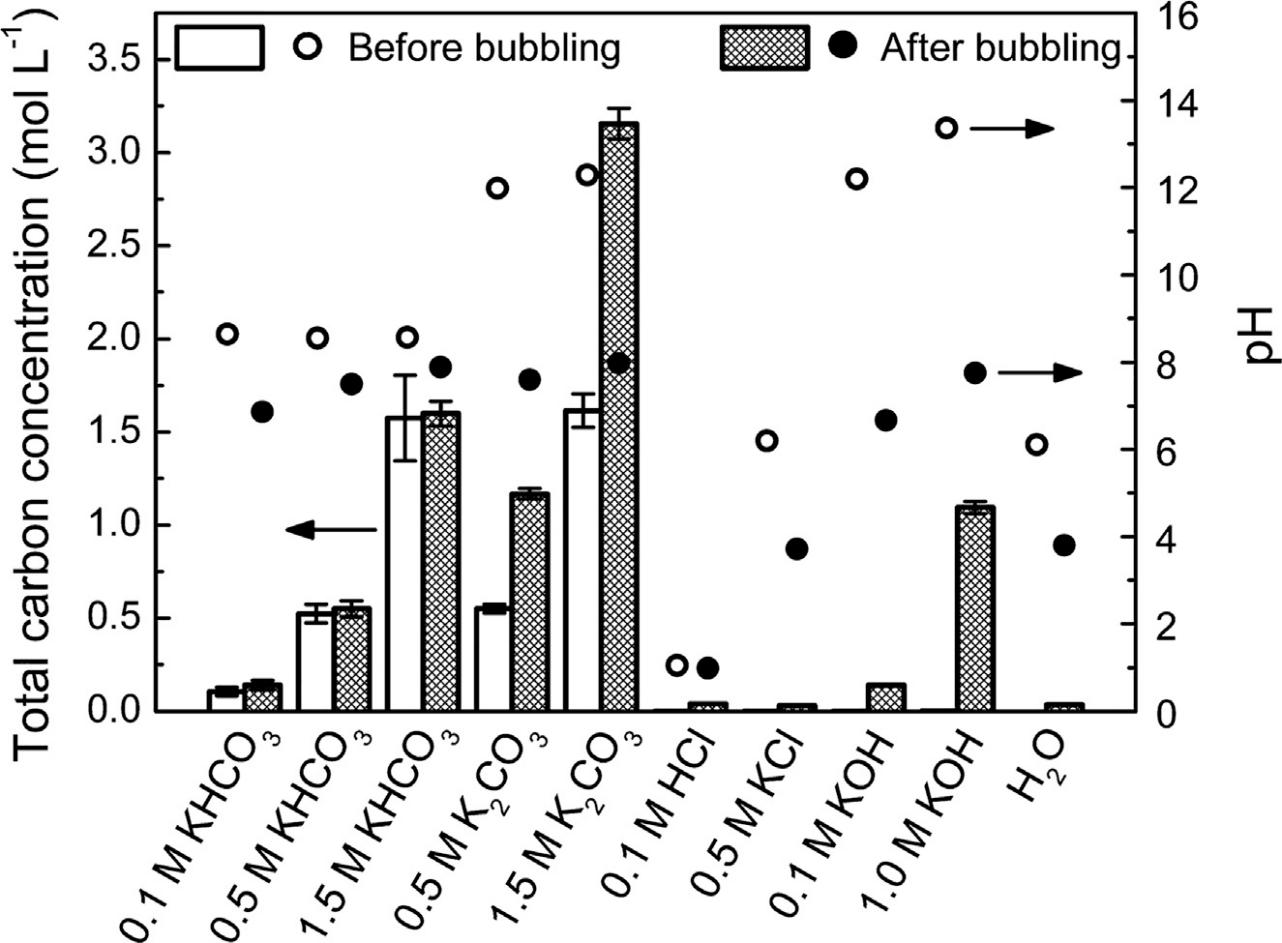
Changes in Total Carbon Concentration and pH Value during Saturation with CO_2_ for Different Commonly Applied Aqueous Electrolytes Reproduced with permission from (Zhong et al., 2015).

In general, an increased CO_2_R selectivity over the HER can be observed with an increase in pH. At pH values (pH > 9) the CO_2_ concentration in the electrolyte is limited due to a shift in the carbonic acid equilibrium to CO_3_^2−^ (pK_a_(HCO_3_^-^) = 10.33, see Table 2). In this context, the CO_2_ supply into solution needs to be considered also. Depending on the buffer capacity (see section Anion Effect), pH value, electrolyte species, and cell operation mode (batch mode versus continuously CO_2_-supplied flow cell) the CO_2_ saturation impacts the local environment at the working electrode (WE). Zhong et al. (Zhong et al., 2015) have investigated the changes in the pH value through the carbonic acid equilibrium during the saturation of different electrolytes with CO_2_. Figure 4 displays the measured total carbon concentration and the pH value before and after saturation for different commonly applied electrolytes. For KHCO_3_ electrolytes with concentrations of *c*(KHCO_3_) = 0.1–.5 M the pH value shifts from pH ≈ 9 down to pH ≈ 7, 7.5, and 8, respectively. Even more significant are the shifts in pH value for 0.1 M and 1 M KOH, dropping from pH ≈ 12.5 and pH ≈ 13.5 to pH ≈ 7 and pH ≈ 8, respectively.

**Table 2:** pK_a_ Values at T = 25°C for Commonly Applied Buffering Anions Carbonate CO_3_^2−^, Sulfate SO_4_^2^−, and Phosphate PO_4_^3−^

Buffering Agent	pK_a,1_	pK_a,2_	pK_a,3_
H_2_CO_3_	3.88	10.33	–
H_2_SO_4_	<0	1.87	–
H_3_PO_4_	2.161	7.207	12.325

pKa values adopted from (Wiberg and Hollemann, 2007).

With the application of GDEs with a continuous CO_2_ supply, the deactivation of CO_2_RR electrocatalysts in favor of the HER at high pH values is not observed in the same manner, as a higher local *c*(CO_2, aq_) can be retained. Dissolved CO_2_ can react at the active site before it is deactivated by the formation of CO_3_^2−^. Consequently, the CO_2_RR can be operated at higher pH values. A variation in pH value between 8.4 and 12 showed no significant impact on the *FE* to formate at current densities between 10 and 250 mA·cm^−2^ in the application of carbon-supported SnO_2_ GDEs in a semi-batch reactor (continuous supply of CO_2_), as reported by Kopljar et al. (Kopljar et al., 2014). Similarly, Dinh et al. (Dinh et al., 2018) investigated Cu-supported GDEs in 1–10 M KOH electrolytes in the CO_2_RR to ethylene. The CO_2_RR in a discrete, thin catalyst layer enabled a larger CO_(ads)_ coverage on the catalyst, favoring the key step in the dimerization to form ethylene, enhancing both activity (Δη = 300 mV between 1 M and 10 M KOH) and selectivity of the reaction.

These results support the notion that operating conditions (CO_2_ saturation, convection, reactor stirring, [effective] diffusion coefficient *D*_eff_ of reactants and products, submerged electrodes, or porous GDEs) can significantly impact the actual local reaction conditions. This must be considered when comparing electrolytes in different reaction systems. In addition, a distinction between electrolyte (bulk) pH and actual (local) pH at the WE must be made. Several publications (Kas et al., 2015, Varela et al., 2016a, Varela et al., 2016b, Clark and Bell, 2018, Hashiba et al., 2018, Hegner et al., 2018, Resasco et al., 2018) have discussed and expressed the importance of the local pH value in the vicinity of the electrode as a pivotal factor in the CO_2_RR. Smith et al. (Burdyny and Smith, 2019) recently emphasized the application of novel catalysts at commercially relevant reaction conditions, as not only the optimization of the electrolyte but also the catalyst performance drastically depends on the local environment of the electrode. This includes the application at large current densities as well as the application of GDEs in continuously operated flow cells (Ahangari et al., 2019). An overview of GDEs and electrolytes applied in aqueous CO_2_RR is given in Table 1.

### Anion Effect

Supporting electrolyte anions serving as buffer agents (e.g., HCO_3_^-^) have shown to affect the local pH value (Dunwell et al., 2017, Hashiba et al., 2018, Resasco et al., 2018) by confining the increase in alkalinity due to the formation of OH^−^ during the CO_2_RR. Therefore the pH gradient and CO_2(aq)_ concentration gradient between the inner Helmholtz plane at the electrode surface and the bulk electrolyte is reduced compared with the unbuffered system, which reduces the polarization overpotentials and increases the local *c*(CO_2(aq)_). The pK_a_ value for applied electrolyte buffering anions are given in Table 2. At a solution pH equal to the pK_a_ value of a buffer, the concentration of the acid and the corresponding base are equal and the buffer capacity (during addition of an acid or base) is maximized.

In comparison, between different electrolyte anions that can act as pH-buffering agents, the bicarbonate electrolyte has the unique ability to serve as a CO_2_ reservoir. HCO_3_^-^ is the most commonly applied electrolyte anion, as it was shown to enhance the CO_2_ reduction rates by effectively increasing the local CO_2_ concentration through its equilibrium with CO_2(aq)_, as reported, e.g., for the reduction to CO on Au (Dunwell et al., 2017). Computational efforts to model the local pH value (Gupta et al., 2006, Hegner et al., 2018) as well as to directly assess it through *in situ* measurements (Clark and Bell, 2018, Dunwell et al., 2018) can therefore help to get a better understanding of how the pH value affects a specific CO_2_RR system. Dunwell et al. (Dunwell et al., 2018) have recently reported the indirect measurement of the surface pH utilizing *in situ* attenuated total reflectance surface-enhanced infrared absorption spectroscopy (ATR-SEIRAS). The ratio of vibrational bands of CO_3_^2−^ and HCO_3_^-^ species at the surface of an Au film cathode is measured; the local pH value is then calculated through the pH-dependent equilibrium between CO_3_^2−^ and HCO_3_^-^. With this, the authors could show changes in WE surface pH and CO_2_ concentration during the reaction, allowing the elucidation of concentration gradients between bulk electrolyte and WE surface for both stirred and unstirred systems and underline the impact of the buffering capacity of an electrolyte.

Besides pH (buffering) effects, a change in both activity and selectivity is reported with the application of differently sized halides. Several authors have recently investigated this effect in the CO_2_RR at Cu/CuO_x_ (Ogura et al., 2010, Varela et al., 2016a, Varela et al., 2016b, Gao et al., 2017, Dinh et al., 2018, Gao et al., 2018, Gao et al., 2019, Huang et al., 2018, Resasco et al., 2018). The phenomenon is rationalized by the specific adsorption of anions at the electrocatalyst surface, which is increasingly pronounced according to F^−^ < Cl^−^ < Br^−^ < I^−^. Adsorbed anions are linked to an increased adsorbed CO_ads_ coverage on the catalyst surface (Huang et al., 2018), stabilizing the intermediate (Varela et al., 2016a, Varela et al., 2016b, Gao et al., 2017). This accelerates the activity of the electrocatalyst by favoring the protonation of CO_ads_ (see Figure 3) and increasing the *FE* to hydrocarbon products methane (Varela et al., 2016a, Varela et al., 2016b) and C_2+_ products (Gao et al., 2018). In addition, halide anions have been reported to induce morphological changes to the Cu/CuO_x_ surface. A recent review by Gao et al. (Gao et al., 2019) included the electrolyte effects observed at a Cu CO_2_R electrocatalyst. Similarly, an anion effect is reported in the electrocatalytic reduction to CO (Lan et al., 2012, Hong et al., 2017, Nguyen et al., 2018). An increased *FE*(CO) over the HER was reported for larger electrolyte anions at Ag (Lan et al., 2012), Au (Hong et al., 2017), and Zn (Nguyen et al., 2018).

### Cation Effect

In the electrocatalytic reduction of CO_2_ in water, commonly applied electrolyte cations include alkali cations (preferably K^+^ and Na^+^), H^+^, and NH_4_^+^. Several groups have identified a significant shift in the product distribution of CO_2_ reduction products and the competitive reduction of water (HER) related to the nature of cations present. Although most publications are in agreement concerning the trends larger alkali cations have on the product distribution for specific metal electrocatalysts, the exact cause for this disparity is still disputed and different effects are credited to contribute to the observed differences. In 1969, Paik et al. (Paik et al., 1969) conducted the first CO_2_RR focusing on cationic effects. The group reduced CO_2_ at a liquid Hg electrode in LiHCO_3_, NaHCO_3_, and (Et)_4_NHCO_3_ electrolytes at acidic and neutral pH values. The main reaction products were formic acid and H_2_ (through the HER). At a given potential, the measured current density *i* increased according to Li^+^<Na^+^<(Et)_4_N^+^. The reduced overpotentials η for larger electrolyte cations were credited qualitatively to a greater specific adsorption at the cathode, causing a more positive effective potential difference ϕ between the cathode and the bulk electrolyte, favoring the reduction of a neutral species (CO_2_) over the positively charged H^+^, hence suppressing the HER in acidic media. As the higher propensity of large cations for specific adsorption results in a less negative potential at the outer Helmholtz plane, the local H^+^ concentration at the cathode is decreased. The influence of ϕ on the pH gradient between bulk electrolyte and electrode surface was first described by Frumkin (Frumkin, 1933) (see Equation 11).

(Equation 11) $$c_{WE}\left(H^{+}\right)=c_{bulk}\left(H^{+}\right)\cdot e^{-\frac{F\cdot \phi }{R\cdot T}}$$

Therefore the less negative potential reduces the concentration of protons *c*(H^+^) at the WE compared with the more negative potentials, increasing the required reduction potential according to Nernst, Equation 5. Similar effects were observed for other electrocatalysts by other research groups; the product distribution at Cu electrodes during CO_2_RR in bicarbonate solutions toward ethylene and alcohols was investigated by Kyriacou (Kyriacou and Anagnostopoulos, 1993) and Hori, respectively (Murata and Hori, 1991). The experiments showed increased *FE*s promoted by larger cation sizes (Li^+^<Na^+^<K^+^<Cs^+^). In return, the *FE*s for CH_4_ and the HER were increased for smaller cations. The application of an NH_4_HCO_3_ electrolyte leads nearly exclusively toward the HER (Kyriacou and Anagnostopoulos, 1993). The differences in product distribution were ascribed to the higher hydration number of smaller cations, which restricts the specific adsorption of the cations, again increasing the effective potential between electrode surface and bulk electrolyte, favoring the HER.

In 2012, Kenis et al. (Thorson et al., 2012) investigated the effect of cations in the electrocatalytic conversion of CO_2_ to CO at Ag GDEs. Similar to previously reported results, the HER is promoted by more hydrated, smaller cations with a smaller tendency for a specific adsorption on the cathode surface. In addition, the stabilization of anions at the cathode surface by specifically adsorbed cations in the vicinity of the electrode is discussed. A stabilization of the CO_2_^−·^ radical intermediate by larger cations therefore could contribute to the increased *FE*s for larger cations (Thorson et al., 2012). Singh et al. (Singh et al., 2016) recently investigated the electrocatalytic reduction of CO_2_ at both Cu and Ag electrodes, including experimental and computational efforts to explain cation effects. Based on density functional theory calculations by Janik (Mills et al., 2014) and Markovic̀ (Strmcnik et al., 2009), the authors suggest that specific adsorption of cations cannot account for the observed differences, as more negative WE potentials would be required for a specific adsorption. The authors therefore suggest the alkali cations in proximity of the WE are subject to hydrolysis and can therefore act as a pH buffer (see Equation 12). In addition to the increased local pH value through OH^−^ formation as a by-product (or H^+^ consumption, depending on the pH), the pK_a_ values for the hydrolysis of the hydrated alkali cations decrease in proximity of the cathode as the O-H bonds of the water ligands between cation and cathode are increasingly polarized. Based on the pK_a_ value for the hydrolysis of the cation, the local pH increase during the reaction is then buffered.

(Equation 12) $$M^{+}{\left(H_{2}O\right)}_{n}+H_{2}O\;\;⇄\;\;MOH{\left(H_{2}O\right)}_{n-1}+H^{+}$$

In the application of multivalent cations, an increase in CO_2_ reduction rate was found with increasing cation valency by Schizodimou et al. (Schizodimou and Kyriacou, 2012). To further elucidate cation effects, Ayemoba and Cuesta (Ayemoba and Cuesta, 2017) probed the pH value on an Au cathode surface with different alkali bicarbonate electrolytes utilizing ATR-SEIRAS, similar to the application by Dunwell et al. (Dunwell et al., 2018) discussed earlier. Their results show a reduced local pH value for larger alkali cations, therefore being in agreement with the hydrolysis hypothesis. Although they confirm the results by Singh et al., it was also concluded that the effect of a reduced pK_a_ in the vicinity of the electrode on the local pH value were overestimated.

### Electrolyte Concentration

A differentiation has to be made between two types of effects concerning the concentration of the electrolyte. First, the direct effects of the electrolyte concentration need to be considered. The energy efficiency *EE* is a key indicator to judge the economic viability of a process. In an effort to reduce the overall cell voltage *E*_cell_, the voltage drop caused by the electrolyte $\Delta \phi_{solution}$ can be reduced by, e.g., minimizing the electrode distance and increasing the electrolyte conductivity (Verma et al., 2016).

(Equation 13) $$\Delta \phi_{solution}=\Delta \phi_{ohmic}+\Delta \phi_{diffusion}=\int \frac{i_{l}}{\kappa }dx+\sum_{i}\int \frac{F\cdot z_{i}\cdot D_{i}\cdot \nabla \cdot c_{i}}{\kappa }$$

with *i*_l_ being the electrolyte current density, κ the electrolyte conductivity, *x* the position, *F* Faraday's constant, *z*_i_ the charge number, *D*_i_ the diffusion coefficient, and *c*_i_ the concentration of the i^th^ species (Singh et al., 2015).

Increasing electrolyte concentration enhances the conductivity of the electrolyte. Significantly reduced cell voltages have been reported in the literature by increasing the electrolyte concentration, especially at increased current densities (Kopljar et al., 2016, Gurudayal et al., 2017). In addition, the salinity of an electrolyte reduces its CO_2_ solubility (see Figure 1). In diluted electrolyte solutions, the effect of CO_2_ on the salinity can generally be neglected, as other, secondary electrolyte effects have a more prominent impact.

Second, the electrolyte concentration impacts the CO_2_RR through the effect the intrinsic properties of the electrolyte has on the reaction conditions (e.g., an increased pH value through higher concentrated KOH, a higher buffer capacity through an increased *c*(KHCO_3_)). Similar to the effect of pH value on the CO_2_RR, it has proved challenging to investigate the effect of the electrolyte concentration in an isolated manner, as a change in electrolyte concentration also affects the pH depending on, e.g., the electrolyte species, cell operation, and CO_2_ saturation. Both for electrolyte cations (Thorson et al., 2012) and for anions (Ogura et al., 2010, Varela et al., 2016a, Varela et al., 2016b, Gao et al., 2017, Dinh et al., 2018, Gao et al., 2018, Gao et al., 2019, Huang et al., 2018, Resasco et al., 2018), specific adsorption on the catalyst surface is discussed in CO_2_RR literature; the effects were discussed in detail in previous sections. Several publications discuss the effect of electrolyte concentrations on the performance of CO_2_RR catalysts. Although the electrolyte concentration is discussed to affect the *FE*(CO_2_R) both positively (Gurudayal et al., 2017, Hegner et al., 2018) and negatively (Zhong et al., 2017), these effects cannot be explicitly related to a concentration effect of an inert electrolyte, likely relating them to the pH and effect on carbonic acid equilibrium/CO_2_ solubility (Hegner et al., 2018).

## Non-aqueous Electrocatalytic CO_2_ Reduction

### Applied Solvents in CO_2_RR

Table 3 gives an overview of protic and aprotic solvents applied in the CO_2_RR reported in the literature, with aqueous electrolytes being used predominantly. Water as a solvent has obvious advantages such as the low price, its wide availability, and its high sustainability, especially when considering electrochemical processes in which large amounts of solvents are applied and consumed. In contrast, price, toxicity, and safety hazards have to be assessed critically when using organic solvents. In addition, CO_2_RR in aprotic solvents requires the formation of a CO_2_^−·^ anion radical, which generally requires large overpotentials compared with protonated intermediates in the aqueous CO_2_ reduction. Despite those drawbacks compared with aqueous electrolytes, there are plenty of studies on electrocatalytic reduction of CO_2_ in non-aqueous solvents, as the application of organic solvents in CO_2_RR is intriguing for multiple reasons. In addition to a generally higher solubility of CO_2_ in organic solvents compared with water (see Table 3), the use of organic solvents enables different reaction products including value-added C_2+_ products like oxalate (Fischer et al., 1981) and further reduced products including glycolic acid (Gressin et al., 1979), glyoxylic acid. and tartaric acid (Kaiser and Heitz, 1973). Controlling the concentration of water as a reactant in organic solvents provides an additional parameter to direct the efficiency (Díaz-Duque et al., 2015, Rudnev et al., 2016) and product distribution (Tomita et al., 2000) of the reaction. Furthermore, the applicable potential range of the solvent can be enhanced as organic solvents are generally less susceptible to oxidation or reduction compared with water. With the application of aprotic solvents the HER can be avoided entirely, which is much harder to do in aqueous electrolytes. Concerning homogeneous or heterogenized metal complex catalysts, their application can require the use of organic solvents in case they are prone to hydrolysis in aqueous electrolytes. The viscosity η of a solvent affects the rate of mass transfer in solution. The viscosity therefore has implications for both the mass transfer of the CO_2_RR (CO_2_, H^+^ mass transport, product diffusion from active site) as well as the conductivity of the solution, as it impacts the movement of charged electrolyte ions.

**Table 3:** Solvents Applied in the Electrocatalytic Reduction of CO_2_ and Selected Physical Parameters at T = 25°C Impacting the CO_2_RR and the Solvents Potential Applicability

Solvent Applied in Literature	CO_2_ Solubility (Gennaro et al., 1990, Lorimer et al., 1992, Hansen, 2007), *c*/mmol·L^−1^	Viscosity (Izutsu, 2002), η/mPa·s	Relative Permittivity (Izutsu, 2002), ε_r_/-	Donor Number (Izutsu, 2002), *DN*/kJ·mol^−1^	CHEM21 (Prat et al., 2015) Selection Guide Based on Safety, Health, Environment
Acetonitrile (Aylmer-Kelly et al., 1973, Kaiser and Heitz, 1973, Fischer et al., 1981, Eggins and McNeill, 1983, Ikeda et al., 1987, Desilvestro and Pons, 1989, Christensen et al., 1990, Higgins and Christensen, 1995, Gennaro et al., 1996a, Gennaro et al., 1996b, Tomita et al., 2000, Lv et al., 2013, Oh et al., 2014, Sun et al., 2014, Berto et al., 2015, Matsubara et al., 2015, Díaz-Duque et al., 2015, Rudnev et al., 2016, Zhu et al., 2016a, Zhu et al., 2016b, Figueiredo et al., 2016, Mendieta-Reyes et al., 2018)	314 ± 6	0.341	35.9	59.0	Problematic
Dimethylformamide (Gambino and Silvestri, 1973, Lamy et al., 1977, Amatore and Savéant, 1981, Fischer et al., 1981, Goodridge and Presland, 1984, Gennaro et al., 1996a, Gennaro et al., 1996b, Oh et al., 2014, Berto et al., 2015, Kai et al., 2017, Shi et al., 2017)	194 ± 14	0.802	36.7	111.4	Hazardous
Dimethyl sulfoxide (Haynes and Sawyer, 1967, Eggins and McNeill, 1983, Ikeda et al., 1987, Welford et al., 2001, Shi et al., 2017)	131 ± 7	1.99	46.5	124.8	Problematic
Hexamethylphosphoramide (Gambino and Silvestri, 1973, Kaiser and Heitz, 1973)	174 ± 15	3.10	29.6	162.4	Highly hazardous
Methanol (Chang and Rousseau, 1985, Naitoh et al., 1993, Mizuno et al., 1995, Ortiz et al., 1995, Saeki et al., 1995a, Saeki et al., 1995b, Saeki et al., 1996, Mizuno et al., 1997, Eggins et al., 1997, Kaneco et al., 1998a, Kaneco et al., 1998b, Kaneco et al., 1998c, Kaneco et al., 1999a, Kaneco et al., 1999b, Kaneco et al., 1999c, Kaneco et al., 2002, Kaneco et al., 2006a, Kaneco et al., 2006b, Kaneco et al., 2006c, Kaneco et al., 2007a, Kaneco et al., 2007b, Mizuno et al., 1998, Ohta et al., 1998, Aydin and Köleli, 2002, Aydin and Köleli, 2004, Ohya et al., 2009, Murugananthan et al., 2015, Albo and Irabien, 2016)	151 ± 11	0.551	32.7	79.5	Recommended/problematic
Propylene carbonate (Kaiser and Heitz, 1973, Fischer et al., 1981, Eggins and McNeill, 1983, Ito et al., 1985, Ikeda et al., 1987, Ogura and Endo, 1999, Berto et al., 2015, Shi et al., 2017, Shi et al., 2018, Shi et al., 2016, Shen et al., 2018, Shen et al., 2019)	134 ± 9	2.53	66.1	63.2	Problematic
Tetrahydrofuran (Gambino and Silvestri, 1973, Berto et al., 2015)	313 ± 40	0.460	7.6	83.7	Problematic/hazardous
Water	34.5 ± 4.4	0.890	78.4	138.2	Recommended

Mean solubilities adopted from (Gennaro et al., 1990, Lorimer et al., 1992, Hansen, 2007) at p = 101.3 kPa and T = 25°C.

Different reaction products are accessible with the same electrocatalyst only by choice of the solvent. For example, the use of protic or aprotic solvents results in different reaction mechanisms. Proposed reaction mechanisms in protic solvents (e.g., water) include a proton transfer from the solvent to the surface intermediate or an oxygen transfer from a surface intermediate to the solvent. The former takes place in the formation of hydrocarbons, formate, methanol, or ethanol, whereas the latter occurs in the formation of carbon monoxide. Owing to that, a change in the product spectrum is observed in aprotic media.

Comparing results of CO_2_RR executed in different non-aqueous solutions is cumbersome for multiple reasons. There is no standard reference electrode (RE) for measurements in non-aqueous solutions. Comparisons of different solvents in the literature are ever so often not performed at the same potential. As the standard reference potential can differ between the utilized solvents (Lewenstam and Scholz, 2013), e.g., for an Ag/Ag^+^ RE, a constant reference point is not given and the measured potentials cannot be compared between the solvents. This is especially relevant because the product distribution can be highly dependent on the applied potential (Ito et al., 1985) and current density. For the comparison of different solvents and measurements in non-aqueous solutions, IUPAC suggests the indication of potentials versus the redox potential of ferrocene (Gritzner and Kuta, 1984). The ferrocene/ferrocenium (Fc/Fc^+^) redox couple is reversible in most non-aqueous solvents and exhibits only small potential differences between a variety of different solvents.

**Figure 5:**
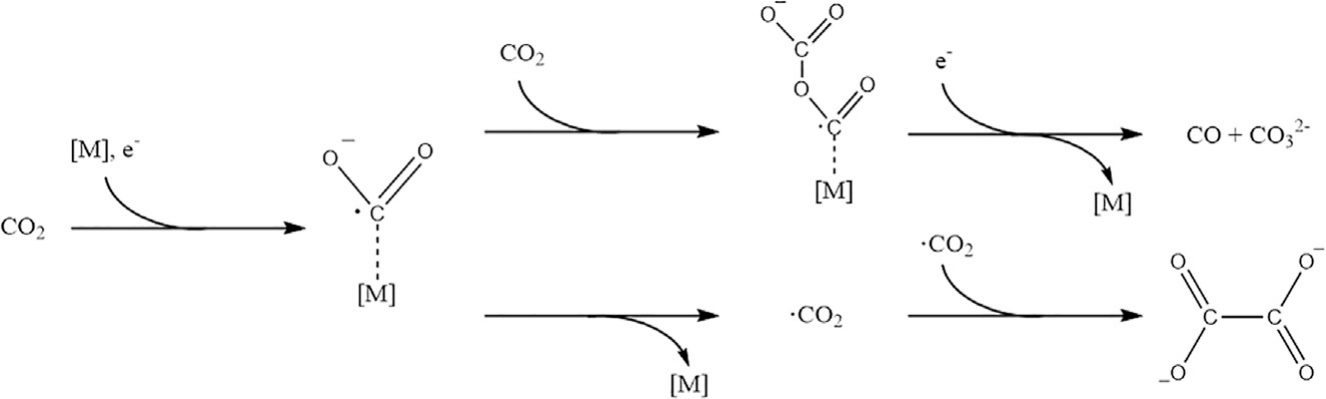
Suggested Reaction Mechanism for the Disproportionation to Carbonate And Carbon Monoxide (Above) and the Dimerization to Oxalate (Below) in Aprotic Media.

Kaiser and Heitz (Kaiser and Heitz, 1973) were the first to investigate the effect different solvents have on the electrocatalytic reduction of CO_2_ in 1973. The main CO_2_ reduction products include oxalate C_2_O_4_^2−^, carbon monoxide CO, and carbonate CO_3_^2−^. In addition to these, residual water in the reactor can lead to the formation of hydrogen, formic acid, and further reduced C_≥2_ products including glyoxylic acid, glycolic acid, and tartaric acid (Kaiser and Heitz, 1973). Their experiments were conducted at current densities between 1 and 20 mA·cm^−2^ in acetonitrile (AN), propylene carbonate (PC), and hexamethylphosphoramide. The results were discussed related to measurements done by Tyssee (Tyssee et al., 1972) in dimethylformamide (DMF) and by Sawyer (Haynes and Sawyer, 1967) in dimethyl sulfoxide (DMSO). Kaiser and Heitz observed that, depending on the applied cathode metal, the main reduction products in an aprotic solvent are either CO and CO_3_^2−^ (strong metal-CO_2_ interaction) or oxalate (weak metal-CO_2_ interaction). This observation is in accordance with their proposed mechanism (see Figure 5) for the formation of oxalate, advancing through a dimerization of two free CO_2_^−·^ radicals. The formation of CO and CO_3_^2−^ is suggested to proceed through a surface-bound CO_2_^−·^ radical. Upon addition of a second solvated CO_2_ molecule and a second electron transfer to the metal-bound CO_2_^−·^ radical complex, an intermediate carbon-oxygen adduct is formed. The adduct consequently disproportionates to form CO and CO_3_^2−^.

Concerning the effect of the chosen solvent, the authors found increased oxalate formation with a decreasing electron donor capability (see donor number, *DN* in Table 3) of the aprotic solvent. The results were explained by the capability of nucleophilic solvents to act as a Lewis base, complexing the slightly positively polarized carbon atom of CO_2_ and therefore inhibiting an electron transfer. Solvents with even lower *DN* (e.g., 1,2-dichloroethane and acetic anhydride, *DN* 0 kJ·mol^−1^ and 44.0 kJ·mol^−1^, respectively, Izutsu, 2002) were found unsuitable due to their low relative permittivity (ε_r_ = 10.4 and 20.7, respectively). A high relative permittivity is necessary for a sufficient dissociation of the added electrolyte salts, providing the conductivity of the solution. Kaiser compares the results based on the assumption that the CO_2_ adsorption at the different applied electrodes (Pb, Hg, CrNiMo-steel) is negligible, as the metals act as a simple electron donor (to form a radical CO_2_^−·^ anion) and not as an electrocatalyst. This is opposed to strongly adsorbing metals, e.g., Pt with a stronger metal-CO_2_ interaction. Ikeda et al. (Ikeda et al., 1987) extended these results by systematically testing various metal electrodes in DMSO, AN, and PC with respect to their selectivity toward oxalate and CO. The authors observed only slight differences ascribed to different amounts of trace water impurities detected in the solvent, indicating that the different solvent properties (see Table 3) do not have a significant impact on the selectivity of the applied metal electrodes.

**Figure 6:**
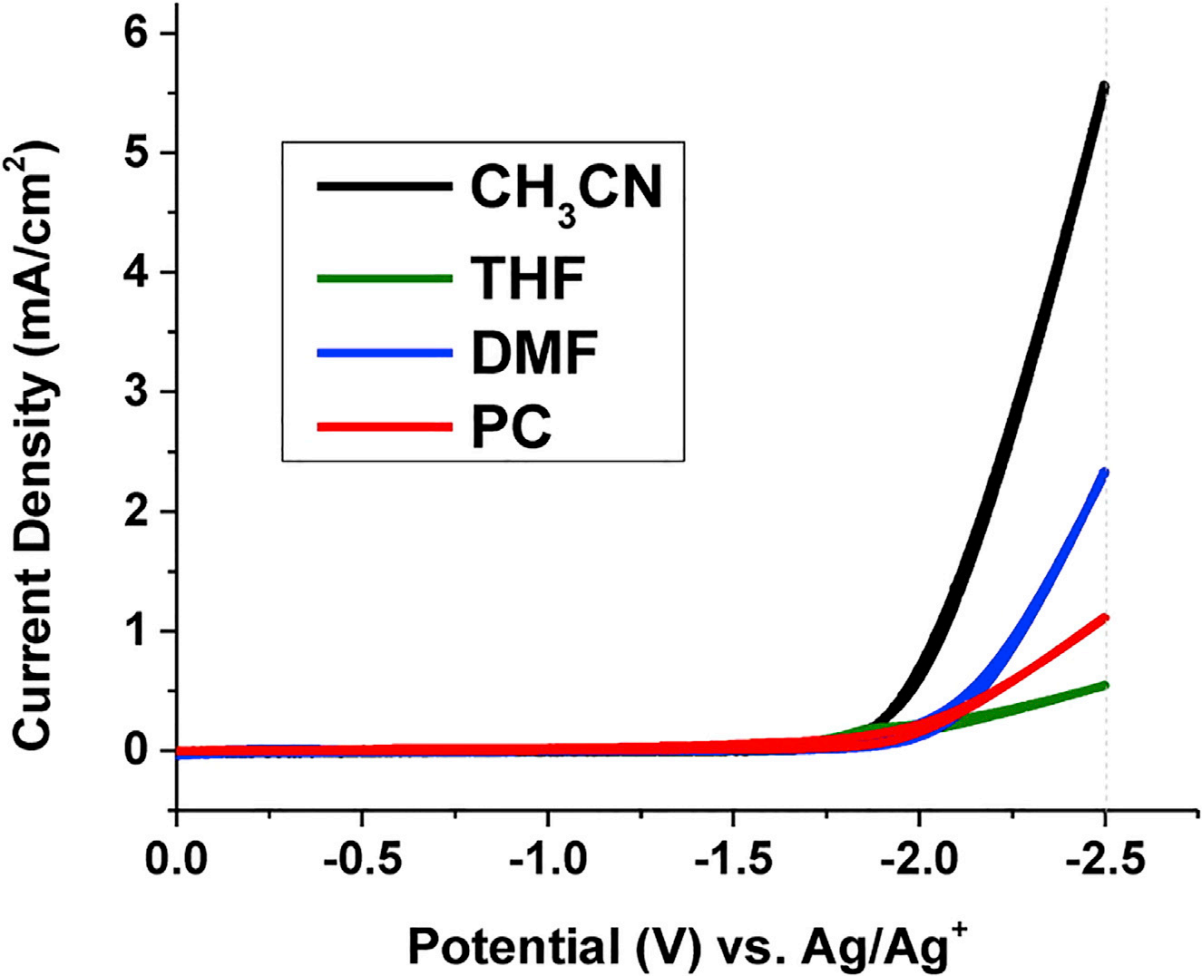
Cyclic Voltammograms Recorded at a Scan Rate of 100 mV·s^−1^ in 0.1 M Bu_4_NPF_6_ in Various Solvents versus Ag/Ag^+^ RE Reproduced with permission from (Berto et al., 2015).

**Figure 7:**
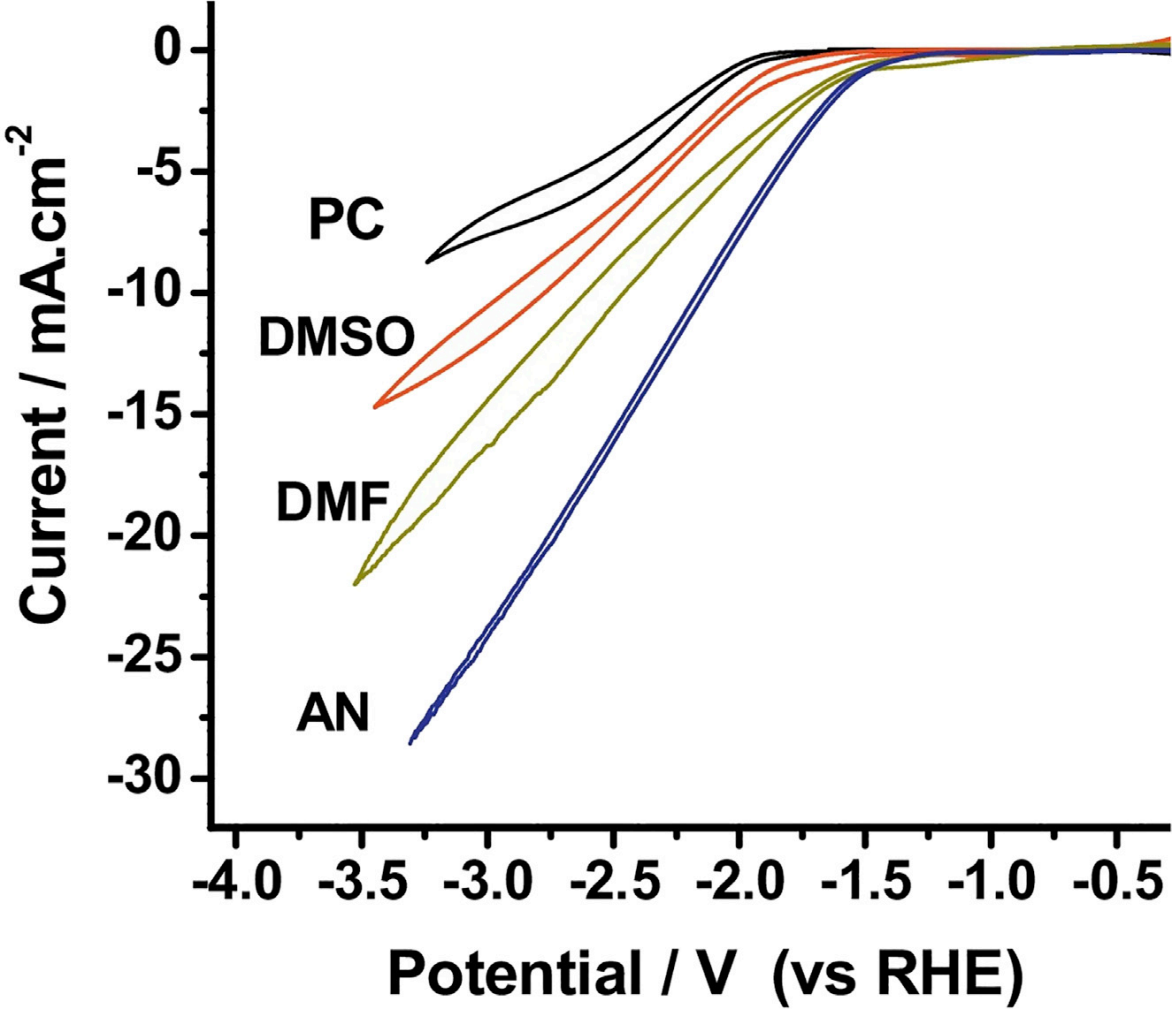
Cyclic Voltammograms Recorded in 0.1 M Bu_4_NClO_4_ in Various Solvents at Au Cathode versus reversible hydrogen electrode (RHE) RE Reproduced with permission from (Shi et al., 2017).

Berto et al. (Berto et al., 2015) compared the onset potentials for the electrocatalytic reduction of CO_2_ on boron-doped diamond electrodes in 0.1 M Bu_4_N^+^ solutions in AN, THF, DMF, and PC (see Figure 6). The predominant reaction products were CO/CO_3_^2−^ and oxalate. The onset potential of CO_2_ reduction is around −1.7 V versus Ag/Ag^+^ for AN and around −2 V for THF, DMF, and PC. The slopes of the Tafel plots differ significantly and were related to the permittivities of the applied solvents. AN, followed by DMF, both with intermediate relative permittivities (ε_r_ = 37.5 and 36.7, respectively) were found to perform at the lowest overpotential η, whereas THF (ε_r_ = 7.58) showed the highest overpotential. Intermediate overpotentials were measured for PC that has the highest relative permittivity ε_r_ = 64.9. Qualitatively similar results were obtained (see Figure 7) by Shi et al. (Shi et al., 2017) for the solvents AN, DMF, DMSO, and PC with PC exhibiting the lowest and AN the highest reduction currents for a given overpotential.

To fully assess the effect different solvent properties have in the CO_2_RR, a deeper understanding of the interactions between solvent, substrate, and intermediate species (solvated or at the electrode surface) is necessary. Summarizing, it seems that single properties such as the relative permittivity, donor number, or the pK_a_ of the solvent seem to be insufficient to describe the effects of the solvents on the activity and product selectivity in the CO_2_RR. This may become particularly interesting when testing new CO_2_RR media utilizing imidazolium-based ionic liquids, which have been proposed to serve as potential co-catalysts in the CO_2_ reduction (Rosen et al., 2011, Snuffin et al., 2011, Sun et al., 2014, Rudnev et al., 2017, Atifi et al., 2018). With a correlation between CO_2_RR performance and key solvent properties, the tailoring of ionic liquid (IL) can be focused on the optimization for a specific CO_2_RR application. ILs enhance the CO_2_ solubility (Cadena et al., 2004) and conductivity, due to their polar nature, and can be tuned to a specific application relatively easily by changing the cation or anion species (Tanner, Batchelor-McAuley and Compton, 2016). They are applied both as solvent or as a supporting electrolyte. In addition, imidazolium (Zhao et al., 2016) and imidazolium derivate cations (Lau et al., 2016) have been shown to act as promoters in the electrocatalytic reduction of CO_2_, which is presumed to stem from the stabilization of the intermediate CO_2_^−·^ anion radical and the consequent reduction of the required overpotential. Sun et al. (Sun et al., 2014) applied 1-ethyl-3-methylimdazolium bis(trifluoromethylsulfonyl)imide as a supporting electrolyte to shift the reaction products from oxalate to CO and CO_3_^2−^. The price for ILs is preventing them from being applied beyond the laboratory scale for now. Further literature on CO_2_RR in ILs can be found in several recent reviews summarizing the IL solvent effects and co-catalytic properties of imidazolium-based supporting electrolytes (Alvarez-Guerra et al., 2015, Lim and Kim, 2017, Sharma and Zhou, 2017, Feng et al., 2018, Faggion et al., 2019).

CO_2_RR in MeOH has extensively been investigated, showing promise for an integrated carbon dioxide capture and conversion technology with already existing CO_2_ capture technologies based on MeOH (RECTISOL process). As a protic solvent with a pK_a_ value only slightly higher than that of water (pK_a_(MeOH) = 17.2, pK_a_(H_2_O) = 14.0, Izutsu, 2002), the reaction products observed in the CO_2_RR with MeOH are similar to those in aqueous solvents. At Cu electrodes, hydrocarbons and alcohols including methane, ethylene, and ethanol are formed (Naitoh et al., 1993, Mizuno et al., 1995, Mizuno et al., 1997, Kaneco et al., 1999a, Kaneco et al., 1999b, Kaneco et al., 1999c, Kaneco et al., 2002, Kaneco et al., 2006a, Kaneco et al., 2006b, Kaneco et al., 2006c, Kaneco et al., 2007b, Kaneco et al., 2007a, Ohya et al., 2009, Murugananthan et al., 2015). Similarly, metals included in the CO-generating group, such as Ag (Saeki et al., 1996, Kaneco et al., 1998a, Kaneco et al., 1998b, Kaneco et al., 1998c), Zn (Saeki et al., 1996), and Au (Kaneco et al., 1998a, Kaneco et al., 1998b, Kaneco et al., 1998c), also produce predominantly CO in MeOH-based electrolytes. However, Pd, which favors the HER over the CO_2_ reduction in aqueous electrolytes, has been reported (Saeki et al., 1996) to produce CO in MeOH as well. Another difference in reaction products is observed in the so-called formic acid group, as methyl formate (Saeki et al., 1996, Kaneco et al., 1998a, Kaneco et al., 1998b, Kaneco et al., 1998c, Kaneco et al., 1999a, Kaneco et al., 1999b, Kaneco et al., 1999c) is detected in the CO_2_RR in MeOH on Pb, Sn, and In. The formation of methyl formate, however, is not the product of a direct CO_2_RR, but rather a consequent condensation reaction between the *in situ*-produced formic acid and the solvent MeOH (Saeki et al., 1996).

### Effect of *c*(H_2_O) in Organic Solvents

The impact of water additions on the product distribution and the activity of metal catalysts has been investigated by several groups. It was found that the CO_2_RR in aprotic solvents is highly sensitive to even small amounts of water (≥46 ppm, Koper et al., Figueiredo et al., 2016), as they impact both the product distribution and the activity of the electrocatalyst.

**Figure 8:**
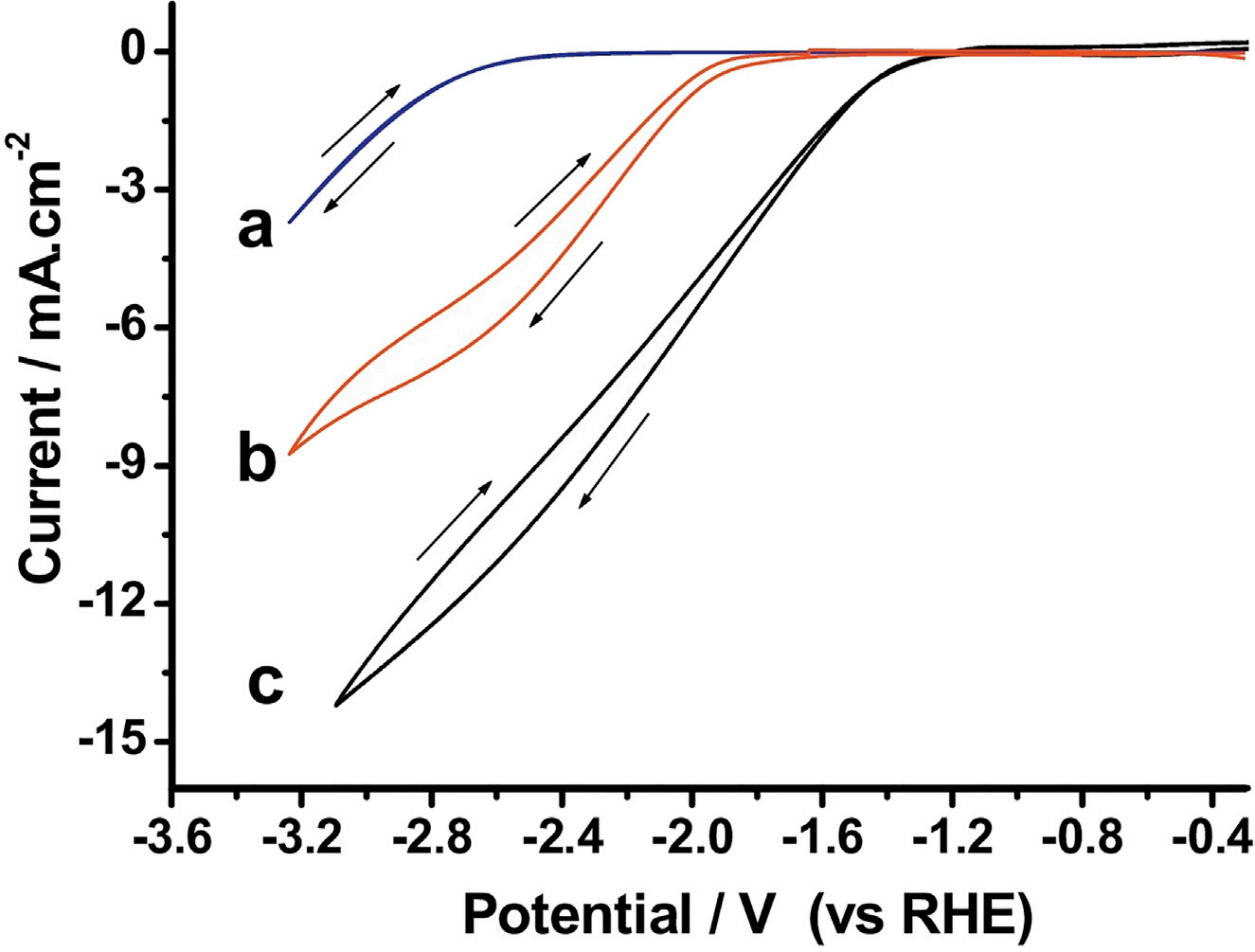
Cyclic Voltammograms at Au versus Reversible Hydrogen Electrode (RHE) in 0.1 M Bu_4_NClO_4_/PC, (a) Ar Saturated, (b) CO_2_ Saturated, and (c) CO_2_ Saturated, 6.8 wt. % H_2_O in Electrolyte Reproduced with permission from (Shi et al., 2017).

**Figure 9:**
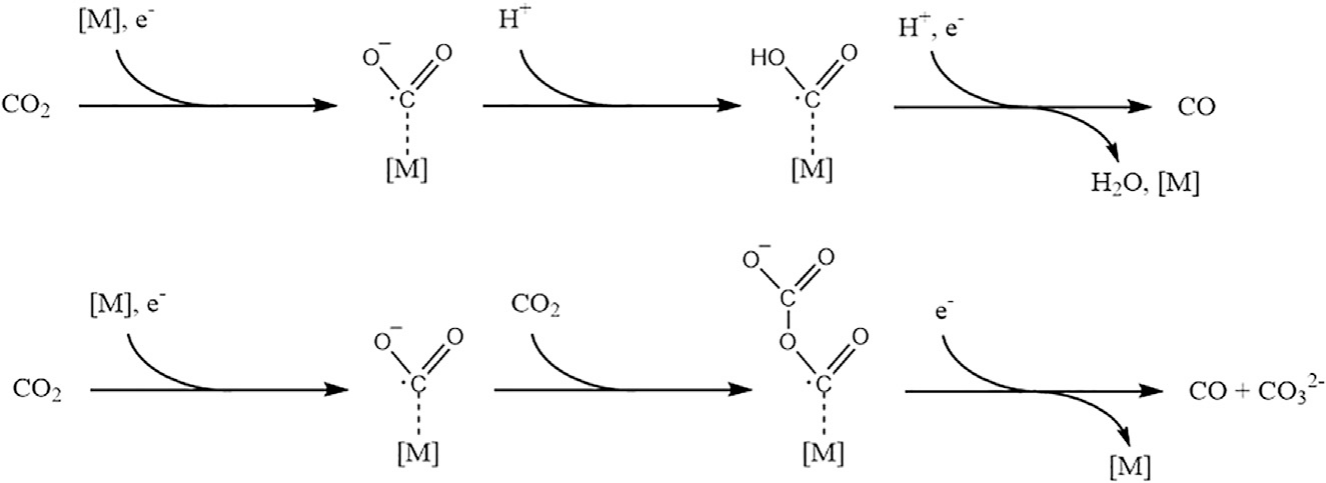
Proposed reaction mechanisms for the electrocatalytic reduction of CO_2_ to CO Top: in the presence of water (Ma et al., 2018), below: in aprotic media (Gennaro et al., 1996a, Gennaro et al., 1996b).

Shi et al. found enhanced CO_2_ solubility and decreased viscosity for a water-saturated 0.1 M Bu_4_NClO_4_/PC solution compared with the water-free electrolyte. During CO_2_RR experiments to CO in PC at Au, an enhanced activity was found (see Figure 8). It is proposed that an alternative reaction mechanism is taking place, shown in Figure 9, where water acts as a proton donor to stabilize the adsorbed CO_2_^−·^ radical anion intermediate (Rudnev et al., 2016).

**Figure 10:**
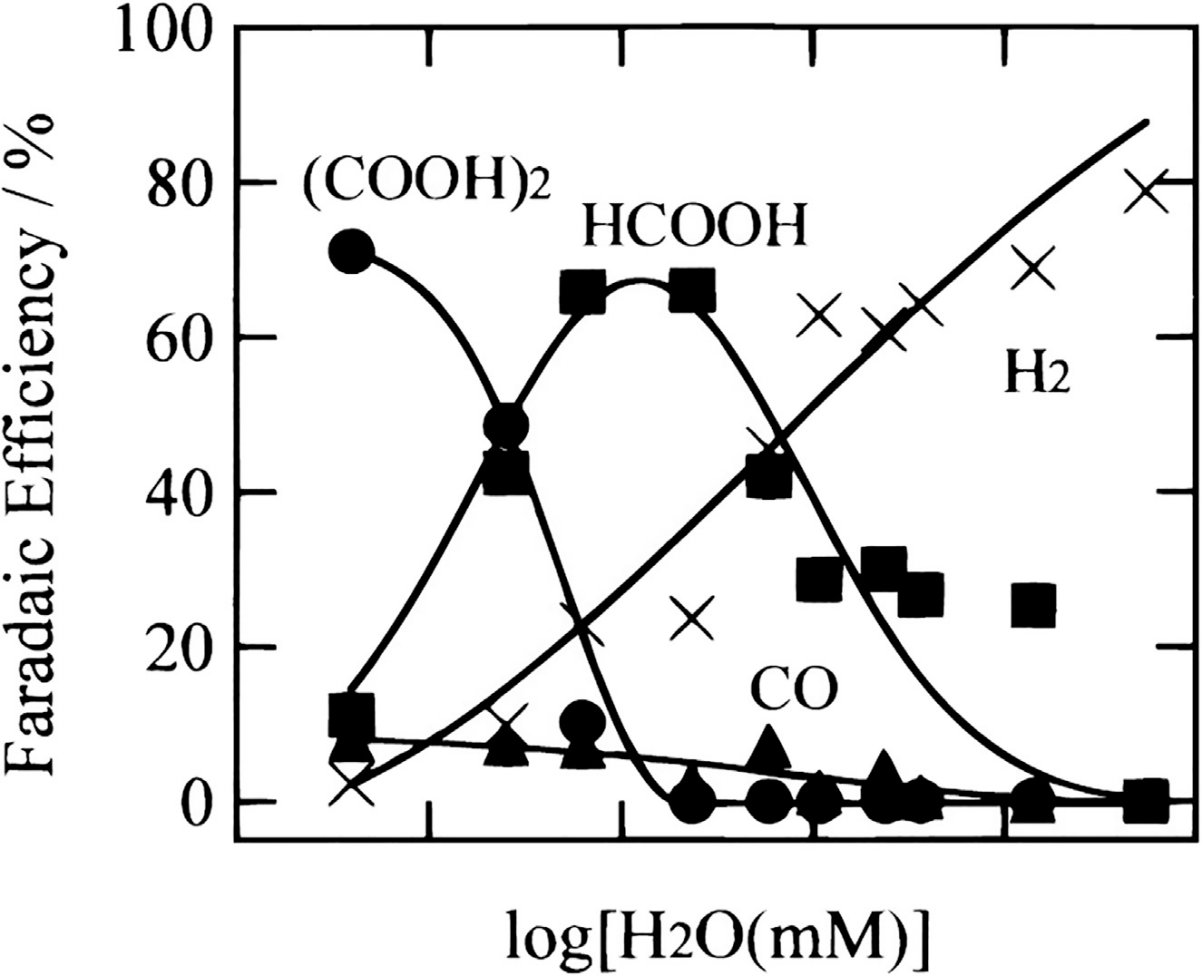
Faradaic Efficiencies for the Products (COOH)_2_, HCOOH, CO, and H_2_ at Different Water Contents in the Electrocatalytic Reduction of CO_2_ at Pt in AN at a Current Density of *i* = 5 mA·cm^−2^ Reproduced with permission from (Tomita et al., 2000).

A similar effect of enhanced activity was observed at nanostructured Cu electrodes in AN (Díaz-Duque et al., 2015), where a H_2_O/AN molar fraction around x = 0.25 showed the highest activity. Tomita et al. (Tomita et al., 2000) investigated the effect of different H_2_O/AN mixtures on the electrocatalytic reduction of CO_2_ at Pt electrodes. Despite a decrease in CO_2_ solubility with increasing water content, the overpotentials for both the CO_2_ reduction and the HER decrease with the water concentration *c*(H_2_O). The formation of oxalate was favored at low water contents (see Figure 10), whereas formate was the major reaction product at intermediate *c*(H_2_O). A maximum *FE* to formate was reached for a concentration of ∼100 mM water. At higher water concentrations (>1 M H_2_O), HER was the predominant reaction.

### Cation Effect in Non-aqueous Solvents

Commonly applied electrolyte cations in protic MeOH are similar to those in aqueous solutions. In aprotic solvents different salts are applied because, in general, the solubilities of alkali metal halides in aprotic solvents are not high enough to provide a sufficient conductivity. Commonly applied supporting electrolytes in aprotic solvents include tetraalkylammonium (R_4_N^+^, e.g., ethyl, butyl) and lithium salts of perchlorates, tetrafluoroborates, hexafluorophosphates, and sulfonates. These salts are more soluble owing to their unpolar nature compared with common aqueous electrolytes. In addition, they are stable in a large potential window, which is required as CO_2_RRs in aprotic solvents typically require potentials more negative compared with aqueous CO_2_RR.

**Figure 11:**
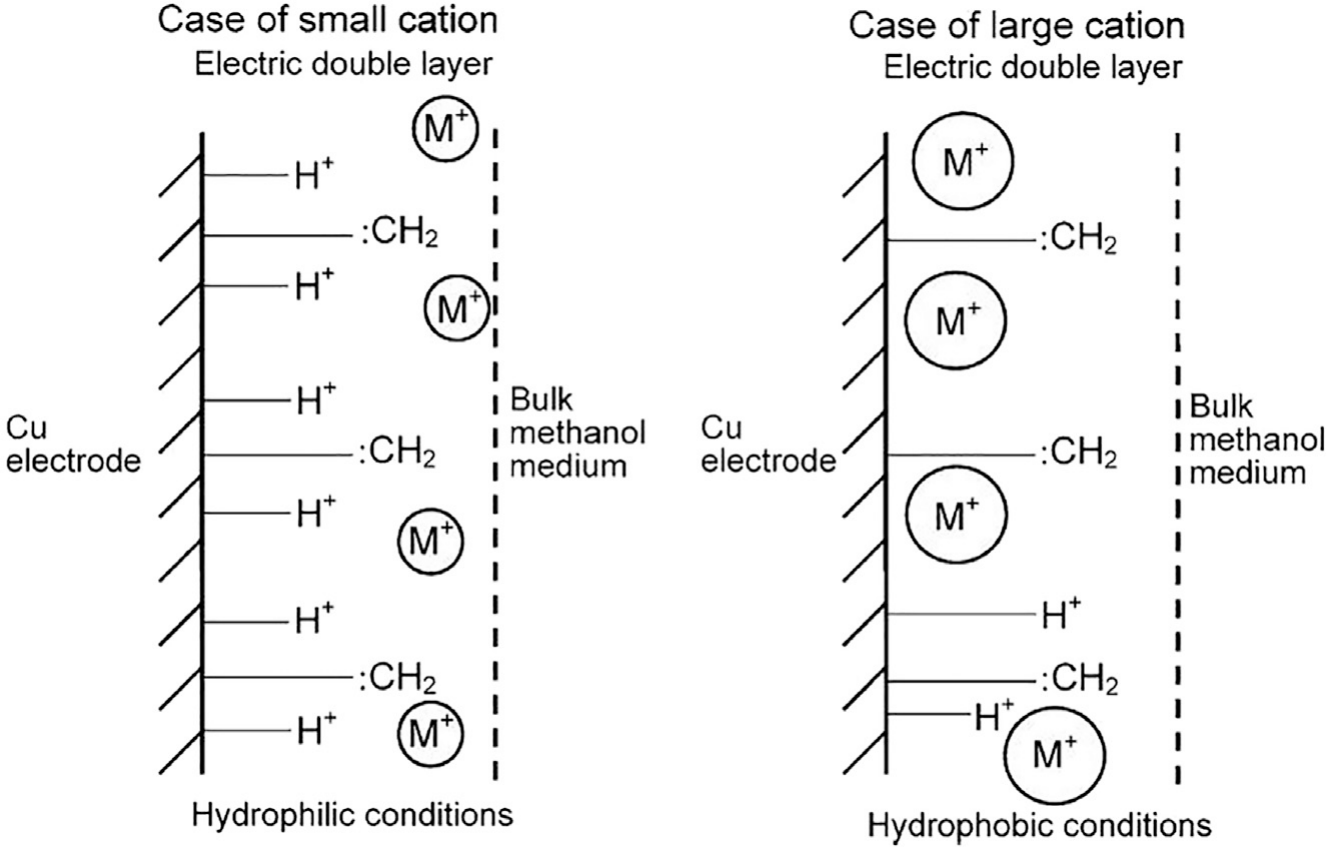
Schematic of Hydrophilic (Protons, Small Cations) and Hydrophobic (Large Cations) WE Environment in the CO_2_RR at Cu Reproduced with permission from (Kaneco et al., 2006a, Kaneco et al., 2006b, Kaneco et al., 2006c).

Kaneco et al. (Kaneco et al., 1999a, Kaneco et al., 1999b, Kaneco et al., 1999c) compared the product selectivity resulting from either KOH or CsOH electrolytes in MeOH on a Cu electrode. Following the trend reported for aqueous electrolytes (see section Aqueous Electrocatalytic CO_2_ Reduction), it was found that the ratio of *FE*s between ethylene and methane was enhanced with Cs^+^ as the electrolyte. Based on these results and similar results in aqueous solutions it was argued that small cations such as Li^+^ and Na^+^ are not directly adsorbing at the electrode surface, owing to their large hydration shell. Conversely, the weakly hydrated, bulky cations are preferentially adsorbed at the cathode (see Figure 11). The rate determining step (RDS), the C-C coupling step in the ethylene formation does not require the presence of adsorbed hydrogen (see Figure 3). Therefore, ethylene formation is favored at lower H^+^ concentrations at the electrode surface, whereas methane formation is dependent on the surface *c*(H^+^).

In 2006, their study was extended toward different electrolyte anions (Kaneco et al., 2006a, Kaneco et al., 2006b, Kaneco et al., 2006c). Only acetate-based electrolytes were found to have a reduced *FE* to CO_2_RR products at Cu electrodes compared with other electrolyte anions (halides, hydroxides, thiocyanates, perchlorates).

### Role of R_4_N^+^ Cations in CO_2_RR

**Figure 12:**
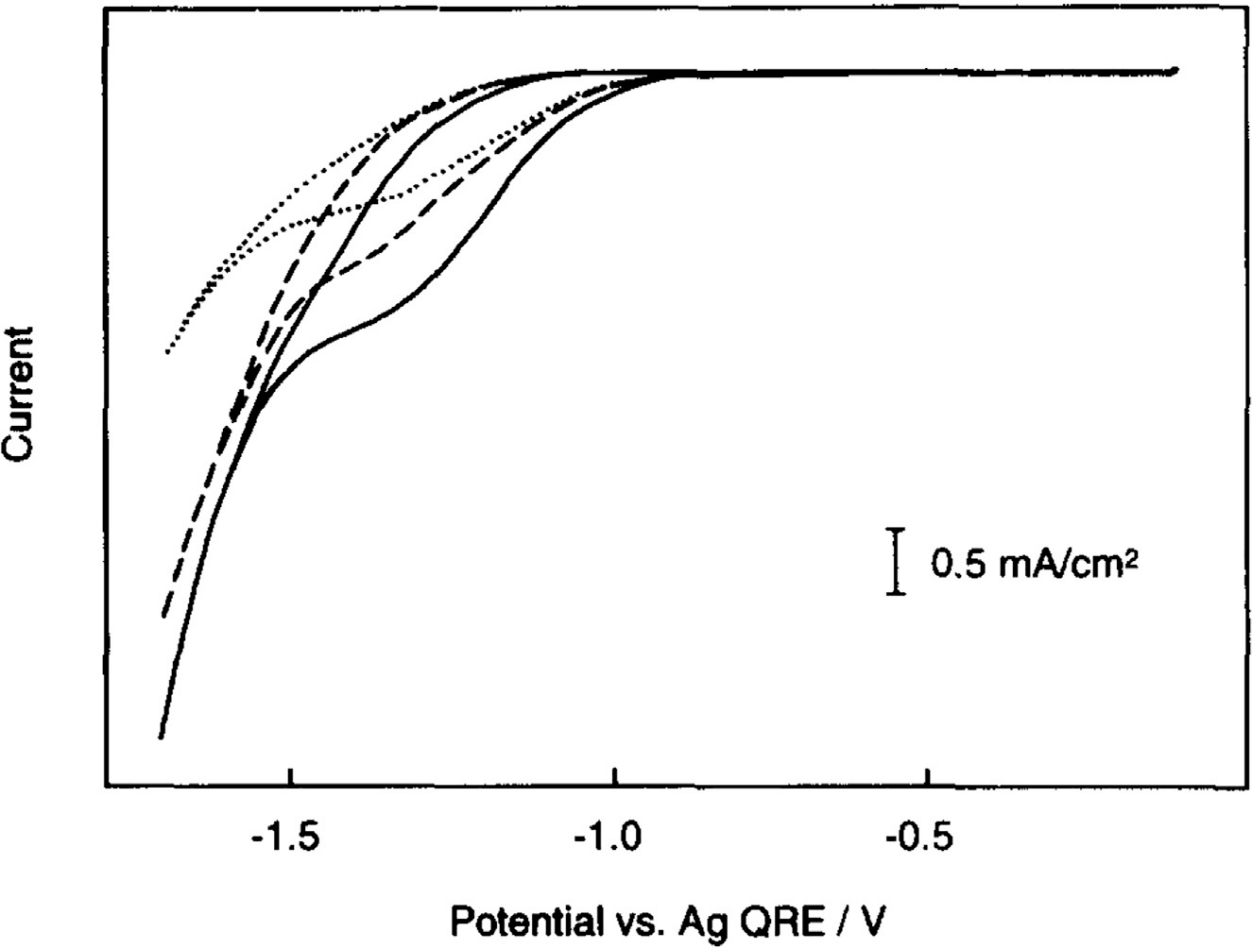
Cyclic Voltammetry Curve of the CO_2_ Reduction in MeOH with Various Concentrations of Bu_4_NBF_4_ (33 mM, dotted; 66 mM, dashed; 0.1 M, solid Line) as a Supporting Electrolyte at a Hg WE (versus Ag Quasi-RE) Reproduced with permission from (Saeki et al., 1995a, Saeki et al., 1995b).

Even though tetraalkylammonium cations are often applied in CO_2_RR in aprotic solvents owing to their exceptional stability and solubility, their impact on the CO_2_RR is disputed. Hori et al. (Tomita et al., 2000) detected no reduction products in a 0.1 M LiClO_4_/AN solution, as opposed to the formation of oxalate, formate, and CO (main products generated on, Pb, Pt, or Au, respectively) when 0.1 M Et_4_NClO_4_ was applied as an electrolyte. It was therefore suggested that the Et_4_N^+^ ion acts as a co-catalyst, either by stabilizing the formed intermediate CO_2_^−·^ anion radical or as a single electron transfer catalyst. Similar results were found during the electrocatalytic reduction of CO_2_ in AN at MoO_2_ by Oh et al. (Oh et al., 2014) and in MeOH at Cu by Saeki et al (Saeki et al., 1995a, Saeki et al., 1995b). In the last-mentioned publication, it was found that the application of a Bu_4_N^+^ electrolyte yielded an increased *FE* to CO under elevated pressure, compared with Li^+^ electrolytes, which showed an increased *FE* to methyl formate and the HER. Figure 12 shows a current-potential curve of the CO_2_ reduction in MeOH with varying Bu_4_NBF_4_ concentrations. It was suggested that the Bu_4_N^+^ ion promotes CO_2_ reduction to CO by either stabilizing the CO_2_^−·^ radical intermediate by forming an ion pair or alternatively stabilizing the surface-bound CO_2_^−·^ by facilitating a hydrophobic environment.

**Figure 13:**
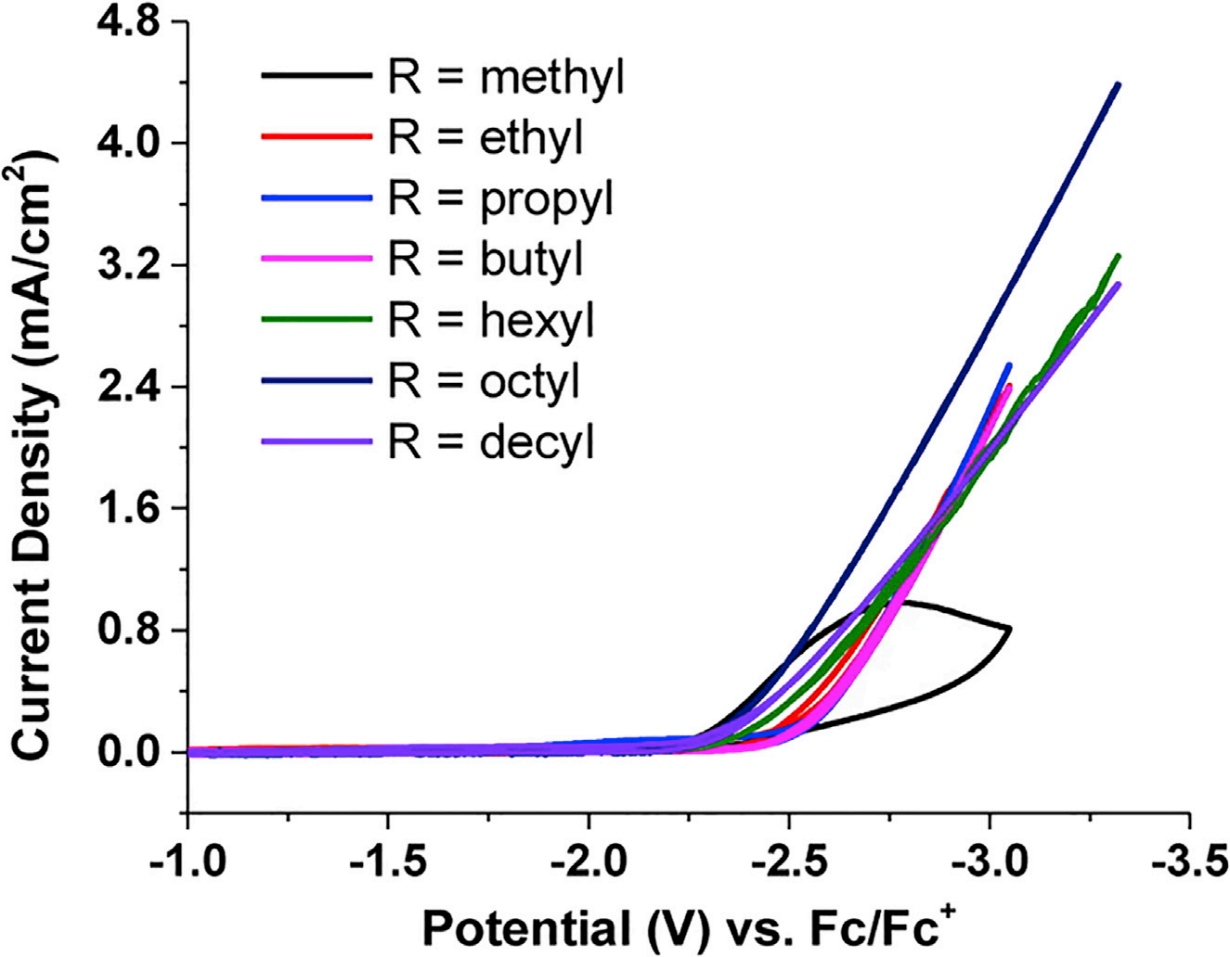
Cyclic Voltammetry at a Boron-Doped Diamond WE (versus Fc/Fc^+^ RE) in CO_2_-Saturated AN with Various 0.05 M R_4_N^+^ Salts Reproduced with permission from (Berto et al., 2015).

**Figure 14:**
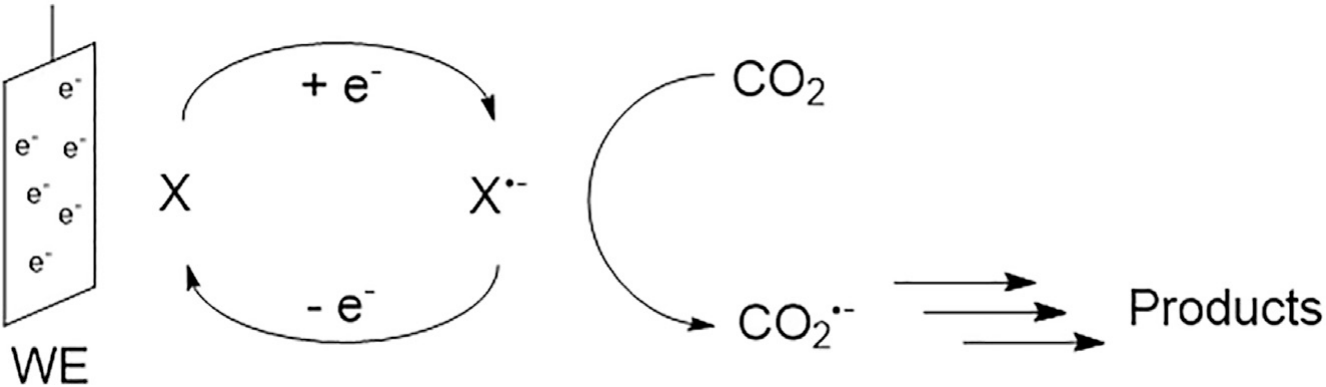
Electrocatalytic Reduction of CO_2_ with a Homogeneous Electron Transfer Catalyst X The electron transfer catalyst is reduced at potentials less negative than the CO_2_/CO_2_^−·^ redox couple.

Berto et al. (Berto et al., 2015) recently discussed the role of R_4_N^+^ in the electrocatalytic reduction in aprotic solvents. The authors argue that the length of the alkyl chain of the R_4_N^+^ cation has no significant impact on the activity of the reaction (see Figure 13), making it unlikely that it is part of the catalytic process. The inability to reduce CO_2_ in a Li^+^-based electrolyte is explained by Li^+^ suppressing the CO_2_ reduction due to the competitive adsorption of the Lewis acid Li^+^ at the electrode surface, forming a hydrophilic layer at the WE. In addition, potentiostatic CO_2_ reduction experiments have shown that the addition of LiClO_4_ to a 0.05 M Bu_4_NClO_4_ in AN electrolyte reduces the measured current and can even arrest it completely (Berto et al., 2015). Setterfield-Price et al. (Setterfield-Price and Dryfe, 2014) reached the same conclusion in an N-methylpyrrolidone-based CO_2_ reduction setup. Their results are supported by surface-enhanced Raman spectroscopy, revealing the formation of deactivating inorganic salts such as LiOCO_2_ and Li_2_CO_3_ at the cathode surface. Another indication that is not serving as an electron mediator is the product distribution observed in aprotic solvents. As shown in Figure 5 the dimerization of two CO_2_^−·^ radical anions take place not on the electrode surface (as the disproportion to CO and CO_3_^2−^ does) but in the bulk of the electrolyte, as proposed by (Costentin et al., 2013). With the application of electron transfer catalysts (aromatic nitriles and esters), as observed by Savéant et al. (Gennaro et al., 1996a, Gennaro et al., 1996b) in DMF, a shift in the product distribution is observed in addition to a reduced overpotential. The use of homogeneous catalysts (see Figure 14) allows for an exclusive formation of oxalate, as the CO_2_ is not directly reduced at the WE, but in the electrolyte. No such shift has been reported for the application of R_4_N^+^ salts as electrolytes.

## Conclusions, Challenges, and Future Directions

The activity and selectivity of electrocatalysts applied in the electrocatalytic CO_2_ reduction are highly dependent on the local environment at the cathode surface. This includes the local CO_2_ concentration, the pH value, and the concentration of the electrolyte. Novel electrocatalysts and electrolyzers are often developed and tested at low current densities. With the goal of upscaling an electrocatalytic process and applying it in industry (Sánchez et al., 2019), it becomes imperative to focus on the development and testing of catalysts and electrolyzers under conditions relevant for their application. At high current densities (at least 200 mA·cm^−2^) and with the application of GDEs, mass transport limitations and their implications (low local CO_2_ concentrations, increased pH values) need to be considered in the development of the electrochemical process. The supporting electrolyte impacts the conditions in the vicinity of the WE (e.g., through the blocking of active sites, influence on the local pH value, and carbonic acid equilibrium through buffering), which deviate from the bulk conditions and between different supporting electrolytes. To get a better understanding of the governing effects the electrolytes have on the CO_2_RR spatially resolved *in situ* measurements (e.g., ATR-SEIRAS) and computational modeling have shown to help in the assessment of the local environment at the active site under real reaction conditions. With a profound understanding of the governing effects and the operational window of a CO_2_ electrolyzer, an optimization for the cost/performance of the system can be made.

The application of GDEs in flow cells allowed CO_2_RR at high current densities under alkaline conditions (e.g., with KOH as an electrolyte for CO_2_ reduction to CO and ethylene, see Table 1 for references), avoiding an increased HER. This is achieved by supplying gaseous CO_2_ during the reaction, allowing the reduction at high current densities even at reduced CO_2_ solubilities. In addition, the application of a continuous process allows a better control of CO_2_ and electrolyte dosing, monitoring the local reaction conditions at the electrode surface. The electrolyte is currently not the main focus in the process of the commercialization of CO_2_RR as required targets concerning the *FE* and *EE* at competitive current densities have yet to be achieved. Still, the product separation and efficient recycling of the electrolyte will have to be managed and the cost, environmental impact, and availability need to be considered in the process of upscaling (De Luna et al., 2019). Another promising approach in aqueous CO_2_R is the application of conductive membranes as solid electrolytes (not discussed in this review), as applied in fuel cells. Although they currently lack long-term stability, by feeding humidified gaseous CO_2_, high current densities comparable to liquid electrolyte systems can be achieved (Li et al., 2016, Weekes et al., 2018).

The application of non-aqueous solvents in the CO_2_RR offers the potential of targeting alternative reaction products like oxalic acid or methyl formate. The advantage of higher achievable limiting current densities due to an increased CO_2_ solubility have been demonstrated for MeOH, especially at reduced temperatures and elevated pressures. In the CO_2_RR in aprotic solvents, generally higher overpotentials are required as the CO_2_ reduction proceeds through the highly energetic CO_2_^−·^ radical anions as opposed to protonated intermediates in aqueous CO_2_RR. In aprotic solvents commonly applied R_4_N^+^ cations facilitate a hydrophobic environment at the WE, favoring CO_2_ reduction. Li^+^ salts, however, inhibit the CO_2_RR (Setterfield-Price and Dryfe, 2014, Berto et al., 2015) by forming a film on cathode surface. Although some publications propose an increased *FE* to CO between CO and C_2_O_4_^2−^ with an increasing basicity of the applied solvent, the exact role of the solvent in the CO_2_RR is not fully understood. Further research is needed to elucidate reported differences in activity and selectivity to relate them to single parameters such as the CO_2_ solubility or basicity of the solvent. Furthermore, additions of water have shown to increase the activity of CO_2_RRs and can shift the product distribution. Mostly oxalate is formed at Pt under aprotic conditions, whereas the main product is formic acid at 10 mM < *c*(H_2_O) < 100 mM, as reported by Tomita et al. (Tomita et al., 2000). The formation of aqueous CO_2_R products due to water impurities is a concern in aprotic CO_2_RR. At the laboratory scale, strict aprotic conditions are therefore required to prevent side products. At larger current densities and during upscaling, Fischer et al. (Fischer et al., 1981) have shown that this is less of a requirement as formation of aqueous CO_2_R products (formate, HER) becomes mass transport limited.
